# Critical Significance of the Region between Helix 1 and 2 for Efficient Dominant-Negative Inhibition by Conversion-Incompetent Prion Protein

**DOI:** 10.1371/journal.ppat.1003466

**Published:** 2013-06-27

**Authors:** Yuzuru Taguchi, Arla M. A. Mistica, Tetsuyuki Kitamoto, Hermann M. Schätzl

**Affiliations:** 1 Departments of Veterinary Sciences and of Molecular Biology, University of Wyoming, Laramie, Wyoming, United States of America; 2 Department of Neurological Science, Tohoku University Graduate School of Medicine, Sendai, Japan; 3 Department of Comparative Biology & Experimental Medicine, Faculty of Veterinary Medicine, University of Calgary, Calgary, Alberta, Canada; Creighton University, United States of America

## Abstract

Prion diseases are fatal infectious neurodegenerative disorders in man and animals associated with the accumulation of the pathogenic isoform PrP^Sc^ of the host-encoded prion protein (PrP^c^). A profound conformational change of PrP^c^ underlies formation of PrP^Sc^ and prion propagation involves conversion of PrP^c^ substrate by direct interaction with PrP^Sc^ template. Identifying the interfaces and modalities of inter-molecular interactions of PrPs will highly advance our understanding of prion propagation in particular and of prion-like mechanisms in general. To identify the region critical for inter-molecular interactions of PrP, we exploited here dominant-negative inhibition (DNI) effects of conversion-incompetent, internally-deleted PrP (ΔPrP) on co-expressed conversion-competent PrP. We created a series of ΔPrPs with different lengths of deletions in the region between first and second α-helix (H1∼H2) which was recently postulated to be of importance in prion species barrier and PrP fibril formation. As previously reported, ΔPrPs uniformly exhibited aberrant properties including detergent insolubility, limited protease digestion resistance, high-mannose type N-linked glycans, and intracellular localization. Although formerly controversial, we demonstrate here that ΔPrPs have a GPI anchor attached. Surprisingly, despite very similar biochemical and cell-biological properties, DNI efficiencies of ΔPrPs varied significantly, dependant on location and inversely correlated with the size of deletion. This data demonstrates that H1∼H2 and the region C-terminal to it are critically important for efficient DNI. It also suggests that this region is involved in PrP-PrP interaction and conversion of PrP^C^ into PrP^Sc^. To reconcile the paradox of how an intracellular PrP can exert DNI, we demonstrate that ΔPrPs are subject to both proteasomal and lysosomal/autophagic degradation pathways. Using autophagy pathways ΔPrPs obtain access to the locale of prion conversion and PrP^Sc^ recycling and can exert DNI there. This shows that the intracellular trafficking of PrPs is more complex than previously anticipated.

## Author Summary

Prion diseases are deadly infectious diseases of the brain characterized by accumulation of a pathologic protein (PrP^Sc^) which is derived from the normal prion protein (PrP^c^). Prions replicate by direct contact in a template-directed refolding process which involves conversion of PrP^C^ into PrP^Sc^. Identifying the modalities of this interaction can advance our molecular understanding of prion diseases. Like substrates and competitive inhibitors of enzymes, a conversion-incompetent PrP can inhibit conversion of normal PrP^C^, a phenomenon known as dominant-negative inhibition (DNI). Interestingly, some conversion-incompetent PrPs efficiently cause DNI but others do not, presumably depending on affinity for PrP^Sc^ and integrity of interaction interface. We utilized DNI to characterize the PrP-PrP interaction interface in cultured cells. We created a series of PrPs with internal deletions in the region between helix 1 and 2 and evaluated their DNI. We found an inverse correlation between deletion size and DNI which suggests that this region plays an important role in PrP-PrP interaction. We also found that such PrPs are subject to various cellular degradation pathways and that a fraction of them reaches the intracellular locale of prion conversion. Further investigation of such prion proteins might help elucidating the cellular mechanisms of the PrP^C^-PrP^Sc^ interaction.

## Introduction

Prion diseases or transmissible spongiform encephalopathies (TSEs) are fatal infectious neurodegenerative disorders causing Creutzfeldt-Jakob disease (CJD) in humans, bovine spongiform encephalopathy (BSE) in cattle, scrapie in sheep and goat, and chronic wasting disease (CWD) in cervids [1]–[5]. The major component of the infectious agent in the pathogenesis of these diseases is the β-sheet rich and partially protease-resistant protein denoted PrP^Sc^, derived from post-translational conversion of the α-helical, protease-sensitive cellular prion protein (PrP^c^) [6], [7]. Prions replicate by template-directed refolding of PrP^c^ into pathological PrP^Sc^, a process which is believed to involve a direct physical interaction of these two isoforms [8], [9]. Although there are a number of proteins whose β-sheet rich conformers are associated with diseases [10], prion diseases are unique among them because prions are clearly infectious at the inter-individual level and can exist in many strains with a stable heritage of the strain-specific properties [11]. The molecular and cellular mechanisms underlying these strain-specific features are still enigmatic. Since PrP isoforms have to physically interact, investigating the PrP-PrP interactions, either PrP^Sc^-PrP^Sc^ or PrP^c^-PrP^Sc^, will provide important information on the molecular mechanisms of prion propagation and delineate new molecular targets for intervention in prion diseases. PrP^Sc^-PrP^Sc^ interaction is important because prion infectivity is enciphered in the structure of PrP^Sc^ [12] and these structures are stably maintained only in the context of PrP^Sc^ oligomers [13], [14]. PrP^c^-PrP^Sc^ interaction is the initial step in the prion conversion process; it therefore affects efficiencies of prion propagation in a host, species barrier phenomena, and high-fidelity inheritance of strain-specific traits [1], [11], [15], [16].

Prion conversion is highly sensitive to mismatches in the primary structures between substrate PrP^C^ and template PrP^Sc^, which can occur in interspecies transmissions or in hosts with polymorphic sites in their *Prnp* genes. Occasionally, even a one-residue mismatch hampers prion propagation and subsequent development of disease [17], [18]. Apparently, such mismatches render the PrP^c^ substrate conversion-incompetent, presumably by impairing its binding to PrP^Sc^ and/or compromising the thermodynamic stability in the conformation as preferred by the PrP^Sc^ template. Of note, besides conversion of itself, a conversion-incompetent PrP occasionally inhibits conversion of a co-existing conversion-competent PrP. This phenomenon is known as dominant-negative inhibition (DNI) or trans-dominant inhibition [17], [19]. Notably, this phenomenon seems also to be of importance in pathologic *in vivo* situations, best exemplified by the naturally occurring protective polymorphisms against scrapie in sheep, CWD in deer, or CJD in humans [20]–[24]. Interestingly, a conversion-incompetent PrP is not synonymous with a DNI-causing PrP; only some conversion-incompetent PrPs exhibit an efficient DNI [17], [25], [26]. Kaneko and colleagues have systematically investigated which substitutions in PrP cause DNI in scrapie-infected mouse cells and postulated a region as an interaction interface with the postulated factor X [17]. DNI was also observed with mutants with internal deletions lacking the secondary-structure components, specifically the first β-strand (B1), the first α-helix (H1) and the second β-strand (B2) [27]. Within a series of consecutive seven-residue insertions in PrP, some of the insertions also caused DNI in various cell-culture systems [26]. Notably, DNI has been recently reported in cell-free systems, suggesting that this mechanism can occur at the level of PrP^c^-PrP^Sc^ interaction, independent of cellular co-factors [25], [28]. A physiological N-terminally truncated degradation product of host-encoded PrP, often referred to as C1 fragment, also exerted DNI in vivo [29]. However, the molecular determinants of the potency of mutant PrP DNI have not yet been defined in detail to our knowledge.

To further investigate PrP^C^-PrP^Sc^ interaction, we utilized the DNI effect for identifying regions of PrP critical for PrP-PrP interaction. We hypothesized that mutant PrPs with efficient DNI must have a high affinity for the template PrP^Sc^ or the substrate PrP^C^, whereas those with inefficient DNI should have lower affinities, and that the difference in affinities originate from the structural integrity and homology in the interaction interface, an analogous logic as postulated for the factor X hypothesis [17]. Unlike observing conversion efficiencies of substrate PrP^C^ which are also affected by thermodynamic stability of the nascent PrP^Sc^, DNI efficiency would mostly depend on the binding affinity of the mutant PrP for PrP^Sc^ or PrP^C^. This allows for a simpler interpretation of results: If mutations in a region affect DNI, the region might compose or be part of the interaction interface. Based on that hypothesis, we focused on the region between H1 and H2 (H1∼H2) which includes the loop between B2 and H2 (B2-H2 loop). Recently, the importance of the B2-H2 loop in PrP^C^-PrP^Sc^ conversion has been highlighted and it was postulated that properties of the B2-H2 loop differ between species and might correlate with species barrier effects [30], [31]. Another recent, although controversial, study provided experimental evidence for a ‘domain-swapping’ event in prion conversion; where N-terminal located subdomains are exchanged between two PrP molecules and thereby B2-H2 loops stretched into β-sheet structures in PrP fibrils which were used as PrP^Sc^ surrogates [32].

To test the importance of the H1∼H2 region in interactions between PrP molecules, we created a series of mutant PrPs with deletions increasing in size in the H1∼H2 portion. Irrespective of deletion size, these mutant PrPs were similar in their biochemical properties (e.g. solubility and PK resistance), subcellular localization, glycosylation profile, and GPI anchoring. However, they were significantly different in DNI efficiencies with an inverse relation between deletion size and DNI efficacy: The larger the deletion the lower was the dominant-negative effect. We also found that the position of the deletions highly affect DNI efficiency. Surprisingly, even mutant PrP with a deletion of the entire region N-terminal to H1∼H2 still showed efficient DNI. These data imply that the structural integrity and the positioning of H1∼H2 are important for PrP-PrP interactions and corroborate the postulated significance of the B2-H2 region for PrP^C^-PrP^Sc^ conversion. Furthermore, we also found that these PrP mutants with internal deletions are trafficked directly from the endoplasmic reticulum to endosomal/lysosomal compartments for degradation through class-III PI3 kinase-dependent pathways, presumably involving autophagic processes. This is highly suggestive of the site where prion conversion occurs and sheds light on the complexity of intracellular trafficking of prion proteins.

## Materials and Methods

### Reagents and antibodies

All buffers and media for cell culture, Hank's balanced salt solution (HBSS) and 10× PBS (pH 7.4) were purchased from Invitrogen Corporation (Carlsbad, CA, USA). Plasmid purification and DNA gel extraction kits were from Omega Bio-Tek (Norcross, GA, USA). Triton X-100 (TX100), deoxycholic acid (DOC), Triton X-114 (TX114), sodium hydroxide (NaOH), N-lauroylsarcosin (sarcosyl), chymotrypsin, and proteinase K (PK) were purchased from Sigma-Aldrich Co., LLC (St. Louis, MO, USA). Guanidine hydrochloride (GdnHCl) was from Promega Corporation (Madison, WI, USA). Site-directed mutagenesis kit was purchased from Agilent Technologies, Inc. (Santa Clara, CA, USA). Tween 20, Bafilomycin A1 (Baf) and acrylamide (40%, 37.5∶1) solution were from EMD Chemicals Inc. (Gibbstown, NJ, USA). Pentosan polysulfate was from Bene-Arzneimittel GmbH (Munich, Germany). 3-methyladenine (3MA) was from Thermo Fisher Scientific Inc. (Waltham, MA, USA). MG-132 was purchased from Cayman Chemical Co. (Ann Arbor, MI, USA). The anti-PrP monoclonal antibody (mAb) 4H11 was described before [33]. The anti-PrP monoclonal antibody (mAb) 3F4 which recognizes residues108–111 was purchased from Covance (Princeton, NJ, USA). Anti-PrP C16-S rabbit mAb, which was raised against the C-terminal part of human H3, was purchased from Novus Biologicals (Littleton, CO, USA). Anti-LAMP1 rat mAb was from BD Biosciences (San Jose, CA, USA). Anti-calnexin (CNX) rabbit polyclonal antibody was from Assay Design's Inc. (Ann Arbor, MI, USA). All the secondary antibodies, DyLight488-conjugated anti-rat IgG antibody, DyLlight594-conjugated anti-mouse IgG antibody and HRP-conjugated or DyLight488-conjugated anti-rabbit IgG (with minimal cross-reactivity with serum proteins from other species) were purchased from Jackson Immunoresearch (West Grove, PA, U.S.A.). The plasmids pEGFP-LC3, -Rab7 and -Rab9 have previously been described [34], [35].

### Cell culture

The mouse neuroblastoma cell line N2a was purchased from American Type Culture Collection (Manassas, VA, U.S.A.) and persistently infected with the mouse prions strain 22L (22L-ScN2a) [36]. A cell population with a high and stable level of PrP^Sc^ was selected by single-cell cloning and used throughout for the experiments described here. The non-infected counterpart (N2a) was prepared by disinfecting 22L-ScN2a cells by pentosan polysulfate (2 µg/ml) treatment over seven passages.

### Site-directed mutagenesis

All primers used in these experiments were ordered from Integrated DNA Technologies, Inc. (Coralville, Iowa, U.S.A.) and are listed in **Table S1**. Mutations were made by site-directed mutagenesis according to the manufacturer's instruction, using a 3F4-epitope (methionine at position 108 and 111) tagged mouse *Prnp* gene cloned into pcDNA3.1(+) as template; therefore all ΔPrPs were 3F4-epitope tagged. Created mutations were verified by sequencing from both strands at Clemson University Genomics Institute (Clemson, SC, U.S.A.).

### Transient transfection for evaluation of expression levels or DNI effect of ΔPrPs and preparation of lysates for immunoblot analysis

For evaluating the expression levels of ΔPrPs, N2a cells on 24-well culture plates were transiently transfected with 0.32 µg/well of each ΔPrP construct. For evaluation of DNI effect, 22L-ScN2a cells on 24-well culture plates were transiently co-transfected with 0.25 µg/well each of (3F4)MoPrP and ΔPrP (total DNA amount 0.5 µg/well; 1∶1 molar ratio). For transfection of cells on 6-well culture plates, 4-fold larger amounts of plasmid and transfection reagent per well than for 24-well plates were used. 22L-ScN2a or N2a cells were plated on 24-well or 6-well culture plates and transfected with plasmid DNA with Lipofectamine LTX Plus kit (Invitrogen) according to the manufacturer's instruction. 2-mercaptoethanol was added to the culture medium (final concentration 50 µM) during transfection. Cells were kept in medium containing plasmid and transfection reagent for 24 hours and then harvested or the old medium was replaced with fresh full medium without plasmid until harvesting. For evaluating effects of various chemical compounds on ΔPrP levels, the old medium was replaced with fresh full medium with either DMSO (0.15%), bafilomycin A1 (120 nM), 3MA (10 mM) or MG132 (10 µM) at this point and the transfected cells were incubated further for 7 hours before harvest. Cells were harvested with phosphate-buffered (pH 7.4) 0.5% TX100, 0.5% DOC (TX100/DOC) lysis buffer, 40 µl/well for 24-well and 300 µl/well for 6-well plate. After removal of nuclear debris by centrifugation (microcentrifuge Eppendorf AG, Hamburg, Germany) at 21,130 g for 1.5 minutes, the supernatant was transferred to another tube as TX100/DOC postnuclear lysate. For samples tested for protease-resistant PrP cores, lysates were digested with chymotrypsin (at indicated concentrations) or proteinase K (PK) (25 µg/ml) at 37°C for 30 minutes. Digestion was stopped by addition of Pefabloc (Roche Applied Science, Indianapolis, IN, U.S.A.) at 2 mM, and 1/4-volume of 5× sample buffer (5×SB; 12% SDS, 250 mM Tris-HCl, pH 7.1, 40% glycerol and bromophenol blue). Finally, lysates were boiled for 10 minutes at 95°C.

### SDS-polyacrylamide gel electrophoresis (SDS-PAGE) and immunoblotting

Samples were resolved on 10–12.5% SDS-PAGE gels and electrotransferred to PVDF membranes (Milipore, Billerica, MA, U.S.A.) by semi-dry blotting method. PVDF membranes were blocked with 5% Blotto (non-fat dry milk from Bio-Rad., Hercules, CA, U.S.A.) in tris-buffered saline supplemented with 0.1% Tween 20 (TBST) for 30 minutes and then incubated with anti-PrP antibodies 3F4 (mAb; 1∶10,000 dilution), C16-S (mAb; 1∶5,000) or 4H11 (mAb; 1∶1,000) in 5% Blotto in TBST. Then membranes were washed in TBST, incubated with HRP-conjugated anti-mouse IgG for mAbs 3F4 and 4H11 or anti-rabbit IgG for mAb C16-S, 1∶10,000 in 5% Blotto in TBST for 1 hour, washed again in TBST four times, then the labeled proteins were visualized with Pierce ECL Plus Western Blotting Substrate (Thermo Scientific, Rockford, IL, U.S.A.). For detection, membranes were exposed to X-ray films (Thermo Scientific, Rockford, IL, U.S.A.) and developed. X-ray films were scanned and quantified by densitometry with image processing software Image J (http://rsb.info.nih.gov/ij/). Acquired data were analyzed with statistics software “R” (www.r-project.org) for two-tail paired *t*-tests with *p*<0.05 as significance level. When re-probing with mAb 4H11 was required, the PVDF membrane was incubated in 100% MeOH for 20 minutes to remove bound antibodies. Then, the membrane was washed in TBST to remove residual MeOH, followed by incubation with mAb 4H11 in 5% Blotto in TBST. The following steps including secondary antibody incubation and washes in TBST were as described above.

### Detergent solubility analysis

N-lauryl sarcosyl was added to TX100/DOC lysates from N2a or 22L-ScN2a cells expressing ΔPrPs to a final concentration of 4%. Lysates were ultracentrifuged using an OptimaTL ultracentrifuge (Beckman Instruments GmbH, Munich, Germany) at 100,000 g, 4°C, for 1 hour. The supernatant was carefully removed, transferred to fresh tubes and subjected to methanol/chloroform (MeOH/CHCl3) precipitation (described below). The pellet fraction was washed once with 100 µl of TX100/DOC lysis buffer and ultracentrifuged again at 100,000 g for 15 minutes. After removal of supernatant, the pellet was sonicated in 1× SB (2.4% SDS; 50 mM Tris-HCl, pH 7.1; 8% glycerol; bromophenol blue) and boiled for 10 minutes.

### Methanol/chloroform (MeOH/CHCl_3_) precipitation

First, 1/5-volume of methanol and 4/5-volume of chloroform were added to lysates, followed by rigorous vortexing for 30 seconds. The mixture was incubated on ice for 20 minutes and then centrifuged at 16,100× g, 4°C, for 30 minutes. The denatured proteins make a sheet between the upper phase with MeOH and the lower phase with CHCl_3_. After removal of the upper phase, MeOH of 9-fold volume of the lower phase was added and mixed again. The mixture was centrifuged at 16,100× g, 4°C, for 30 minutes. Subsequent to centrifugation, the supernatant was thoroughly removed and the protein pellet was dried, dissolved in 1× SB and finally boiled.

### Endoglycosidase H (EndoH) or peptide: N-glycosidase F (PNGaseF) digestion

ΔPrPs were digested with endoglycosidase H (EndoH; New England Biolab Inc., Ipswich, MA, U.S.A.) according to manufacturer's instructions. Briefly, first 100 µl of TX100/DOC lysates were subjected to MeOH/CHCl_3_ precipitation. Protein pellets were re-dissolved in 25 µl of 1× denaturation buffer provided with the enzyme and boiled for 10 minutes. Then, 20 µl of deionized water, 5 µl of G5 reaction buffer and 2 µl of EndoH were added and incubated at 37°C for 1.5 hours. After digestion, 1/4-volume of 5× SB was added and boiled for 10 minutes. For PNGaseF digestion, following MeOH/CHCl_3_ precipitation, the pelleted proteins were reconstituted in dilute sample buffer (5× SB diluted with deionized water, 1∶15) and boiled with shaking at 1,400 r.p.m. for 10 minutes. The denatured proteins were supplemented with 1/10-volume of G7 buffer and 10% NP40 attached to PNGaseF (New England Biolab) and then PNGaseF was added and incubated at 37°C for 2 hours. After incubation, 1/4-volume of 5× SB was added and the samples were boiled for 10 minutes.

### Triton X-114 extraction of ΔPrPs

N2a cells expressing ΔPrPs on 6-well culture plates were incubated in PBS with 3 mM EDTA for 3 minutes and then mechanically detached by pipetting. The cell suspension was collected in a 1.5-ml tube and centrifuged at 1,000× g for 5 minutes at 4°C. After the centrifugation, the supernatant was thoroughly discarded and the pelleted intact cells were resuspended in 400 µl of phosphate-buffered (pH 7.4) 2% TX114 lysis buffer (2% TX114; 137 mM NaCl; 2.7 mM KCl; 8 mM Na_2_HPO_4_; 2 mM KH_2_PO_4_) and incubated on ice for 30 minutes, with vortexing from time to time. Subsequently, the cell suspension was centrifuged at 16,100× g, for 2 minutes at 4°C and the supernatant was transferred to another tube as TX114 lysate. Phase separation of TX114 lysates was done by incubating the lysates at 37°C for 10 minutes and centrifugation at 21,130× g for 10 minutes. The aqueous phase was transferred to another tube and subjected to MeOH/CHCl_3_ precipitation. The remaining detergent phase was diluted with 0.1% TX114 wash buffer (0.1% TX114; 137 mM NaCl; 2.7 mM KCl; 8 mM Na_2_HPO_4_; 2 mM KH_2_PO_4_) and subjected to MeOH/CHCl_3_ precipitation. The precipitated proteins were then processed as described above for ‘MeOH/CHCl_3_ precipitation’. For phase-separation after denaturation with GdnHCl, 120 µl of TX114 lysates were mixed with 120 µl of 6 M GdnHCl and incubated at room temperature for 45 minutes. Then, 150 µl of 2% TX114 lysis buffer along with 1,000 µl of 0.1% TX114 wash buffer were added to dilute out GdnHCl, and lysates were subjected to phase separation. After separation, the aqueous phase was transferred to a fresh tube for MeOH/CHCl_3_ precipitation, the detergent phase (∼50 µl) was diluted with 0.1% TX114 wash buffer up to 200 µl and then subjected to MeOH/CHCl_3_ precipitation. The following steps for sample preparation were as above. For the in vitro PIPLC digestion of GdnHCl-treated lysates, after the first phase separation after denaturation with GdnHCl, the aqueous phase containing GdnHCl was discarded and the detergent phase (∼50 µl) was diluted with 550 µl of 0.1% TX114 wash buffer to further reduce GdnHCl concentration to a sufficiently low level for not inhibiting PIPLC activity. After lysates were cleared on ice, 4 µl of PIPLC (2 U/µl) was added to ‘PIPLC+’ samples and both the lysates with or without PIPLC were incubated at room temperature for 2.5 hours. Then, the lysates were subjected to a second round of phase separation and the aqueous and the detergent phases were processed as described above, except that 3 µl of 10 mg/ml bovine serum albumin (BSA) was added to each phase as a carrier before MeOH/CHCl_3_ precipitation.

### Immunofluorescence analysis/confocal microscopy

Cells were plated on glass coverslips placed at the bottom of 24-well culture plates and transient transfection was performed as described above. Next day, the transiently transfected cells on the coverslips were rinsed twice with HBSS and then fixed with 4% paraformaldehyde (USB Corporation, Cleveland, OH, U.S.A.) at room temperature for 30 minutes. After fixation, cells to be permeabilized were treated with 0.2% Triton X-100 in PBS for 10 minutes at room temperature and rinsed with PBS three times. When antigen retrieval by GdnHCl treatment was required, the permeabilized cells were incubated with 6 M GdnHCl for 45 minutes and then the cells were rinsed with PBS four times before incubation with the primary antibody. Permeabilized and non-permeabilized cells were incubated with mAb 3F4 (1∶2,000) in 3% BSA in PBS for 60 minutes on a rocking platform, followed by washing with PBS four times, then incubated with DyLight488-conjugated sheep anti-mouse IgG (1∶1,000; Jackson Immunoresearch, West Grove, PA, U.S.A.) in 3% BSA in PBS for 45 minutes and finally washed in PBS four times. After immunolabeling, the coverslips with cells on them were taken out from the 24-well plates and mounted on slide glass with a drop of Permafluor mountant (Thermo Scientific, Rockford, IL, U.S.A.). When the mountant was dried, samples were analyzed on a laser scanning confocal microscope, Zeiss710 (Carl Zeiss Inc., Thornwood, NY, U.S.A.), in the Robert A. Jenkins Microscopy Facility of the University of Wyoming. Samples were studied with an objective lens, EC Plan-Neofluar 100×/1.3 Oil Pol M27, and the wave length of the excitation lasers were 488 nm for DyLight488 and EGFP and 594 nm for DyLight594. The acquired image data were processed with Image J (NIH, USA). For observation of colocalization of ΔPrPs with LAMP1, to minimize cross-reactivity of anti-mouse secondary antibody to anti-LAMP1 rat mAb cells were first labeled in the following order: mAb 3F4, DyLight594-anti-mouse, anti-LAMP1 and then DyLight488-anti-rat. If needed, transfected cells were treated with bafilomycin A1 for ∼6 hours before fixation to inhibit the degradation of ΔPrPs by lysosomal proteases. Only a single slice at the level of nucleus where the punctate fluorescence was most abundant was used for analysis of co-localization.

### Subcellular fractionation of transfected cells

N2a cells on a 6-well plate were transiently transfected as described above. Twenty-four hours later, the old medium was replaced with fresh medium, with or without bafilomycin A1, and cells were cultured for 6 more hours. Cells were rinsed once with PBS without calcium or magnesium, then incubated for a few minutes in 3 mM EDTA in PBS until cells could be easily detached from the plate by pipetting. Detached cells in PBS were collected in 1.5 ml tubes and centrifuged at 200× g at 4°C for 5 minutes. After removal of supernatant pelleted cells were resuspended in 200 µl of homogenization buffer [8.5% (w/w) sucrose; 4 mM Tris-HCl, pH 7.1; 2 mM EDTA; 30 µg/ml cycloheximide (Sigma Aldrich) and complete protease-inhibitor cocktail (Roche Applied Science, Indianapolis, IN, USA)]. Before homogenization, 10 µl of cell suspension was taken for whole-cell lysate measurement. The rest of the cells were homogenized by passing through a 25G ultra-thin-wall needle (Terumo Medical Corporation, Somerset, N.J.) until >90% of cells were disrupted. The homogenate was centrifuged at 2,000× g for 5 minutes and the supernatant, ∼175 µl, was transferred to another 1.5 ml tube as post-nuclear fraction and mixed well with 240 µl of a 62% (w/w) sucrose solution to a final concentration of ∼42%. The post-nuclear fraction was placed at the bottom of a centrifuge tube and three layers of sucrose solutions, 36%, 33% or 30%, and homogenization buffer were overlaid from the bottom to the top. The gradient was ultra-centrifuged at 40,000 rpm at 4°C for 1.5 hours with a Beckman SW50.1 swing rotor in a Beckman L8-80M ultracentrifuge. After ultra-centrifugation, fractionated organelles were visible as milky-white bands in interphases between the layers and 350 µl was carefully pipetted from each interphase to collect organelles as completely as possible. Collected interphases were first diluted with 300 µl of PBS with 0.01 µg/µl of BSA and then subjected to MtOH/CHCl_3_ precipitation, as described above. Pelleted proteins were dissolved in 1× SB and boiled to make “interphase” samples. For “whole-cell lysate” samples, 10 µl of cell suspensions were mixed with 30 µl of TX100/DOC lysis buffer. After centrifugation to precipitate the cell debris, supernatants were collected in another tube as whole-cell lysates. After addition of 10 µl of 5× SB, they were boiled to prepare “whole-cell lysate” samples.

## Results

### Engineering mutant PrPs with internal deletions and expression in cultured cells

**Figure 1:**
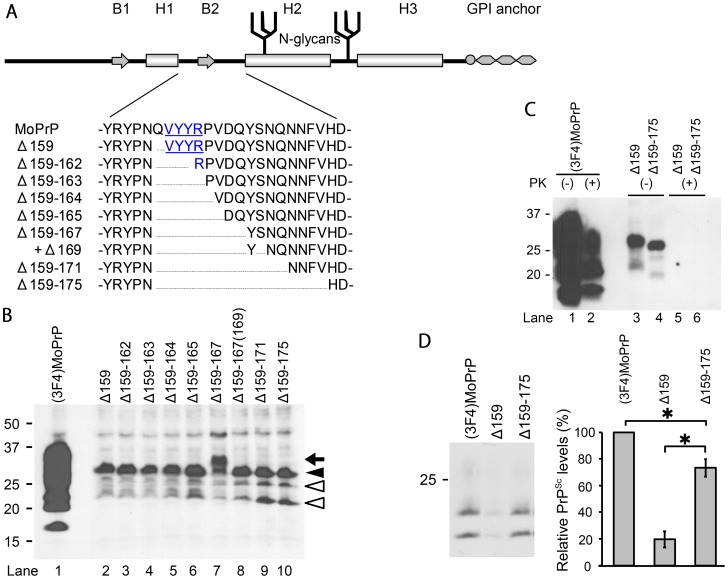
Expression of ΔPrPs and dominant-negative inhibition (DNI) of Δ159 and Δ159–175 in persistently prion-infected cells. **A.** Schematic illustration of prion protein secondary structure elements comprising two β-strands (B1 and B2) and three α-helices (H1–3) and post-translational modifications (two N-linked glycans and glycosylphosphatidylinositol anchor) along with position and extent of deletion of ΔPrPs constructs. Deletions in the H1∼H2 portion have a common N-terminal end (residue 159) and gradually extend into the C-terminal direction. All constructs have a 3F4 epitope tag (methionines at residue 108 and 111). Underlined residues denote B1. **B.** Comparison of ΔPrP expression levels. Representative immunoblot is shown for detection of (3F4)MoPrP and ΔPrPs in transiently transfected N2a cells using mAb 3F4. ΔPrPs are similar in expression level glycosylation appearance, except for Δ159–167 which has an extra fragment larger than the diglycoform (lane 7, arrow). **C.** ΔPrPs are not converted into PK-resistant PrP in prion-infected cells. 22L-ScN2a cells were transiently transfected with (3F4)MoPrP, Δ159 or Δ159–175, and cell lysates digested with PK or not. Immunoblot was done using mAb 3F4. **D.** Pilot study for dominant-negative inhibition (DNI) of Δ159 and Δ159–175. DNI was assessed by co-transfecting 22L-ScN2a cells with (3F4)MoPrP and Δ159 or Δ159–175, respectively, and testing for PK-resistant (3F4)MoPrP. Left panel shows representative immunoblot (mAb 3F4) and right panel the statistical analysis of quantified PK-res levels of a triplicate experiment. Empty pcDNA3.1 plasmid was used as control for co-transfection in lane 1. The error bars indicate standard deviation. *, p<0.05.

**Figure 2:**
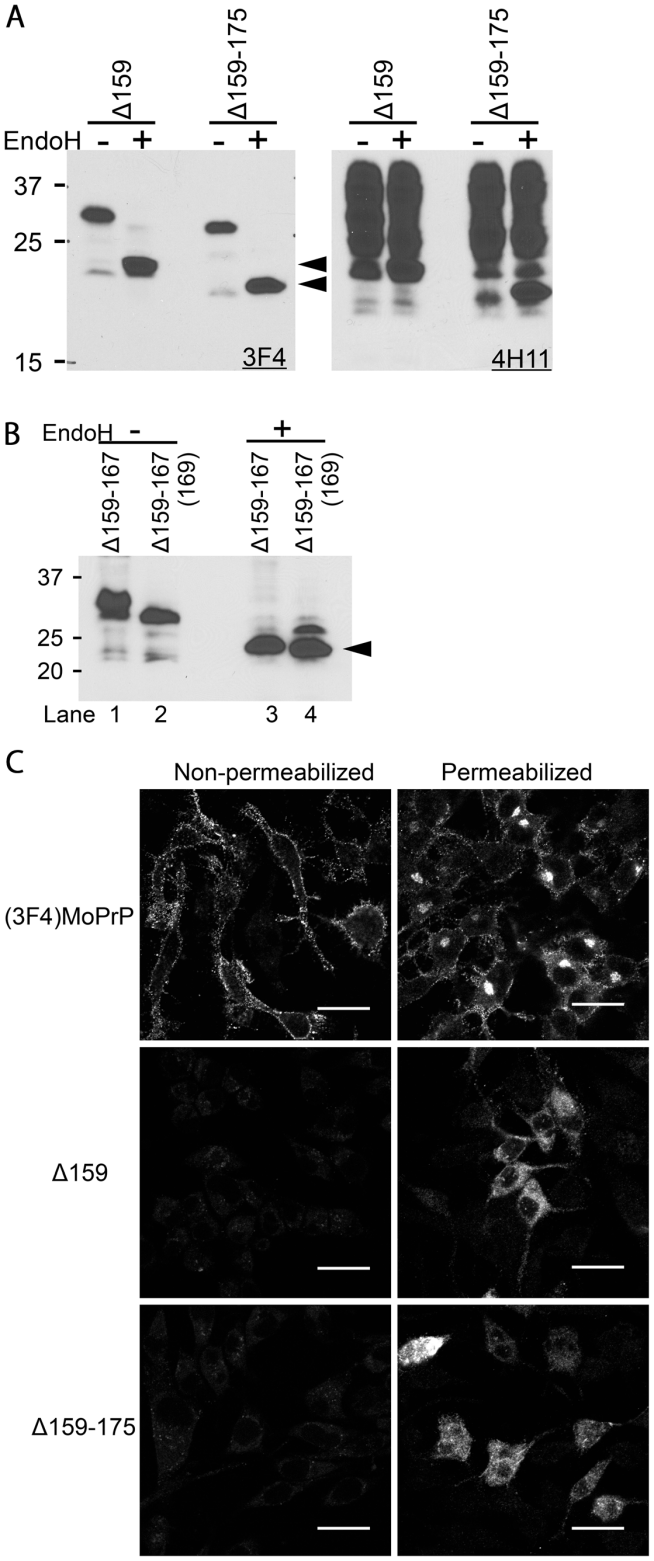
ΔPrPs are non-complex glycosylated and retained intracellularly. **A.** N-linked glycans of ΔPrPs are not complex-type. Immunoblot comparing EndoH- and non-digested lysates of ΔPrP159 and Δ159–175 transfected cells. Left panel was probed with mAb 3F4 mAb, detecting transiently expressed ΔPrPs. On the right panel, the membrane was re-probed with mAb 4H11, detecting total PrP. Arrowheads depict deglycosylated ΔPrPs. **B.** The additional band of Δ159–167 represents a third N-glycan sensitive to EndoH. Upon EndoH digestion, all bands converge into a non-glycoform band (lane 3 and 4). In Δ159–167(169) the third glycosylation site is not present anymore. **C.** ΔPrPs are mainly distributed in intracellular compartments rather than on the cell surface. Confocal microscopy images of N2a cells transiently transfected with (3F4)MoPrP, Δ159 or Δ159–175, either permeabilized (right) or non-permeabilized (left) with 0.2% TX100 after fixation. Cells were immuno-labeled with mAb 3F4. (3F4)MoPrP-expressing cells show bright immunopositivity on the cell surface, whereas ΔPrPs are not observed on the cell surface. Also intracellular staining differs strongly between (3F4)MoPrP and ΔPrP expressing cells Scale bars, 25 µm.

We decided to engineer a series of mutant PrPs (ΔPrPs) with different deletion sizes, starting with a deletion of a single residue (i.e. glutamine 159) immediately after the H1 region, and extending C-terminally towards the H2 region (**Fig. 1A**). We reasoned that such deletion mutants would be more straight-forward for analysis of structure-function relationships than other types of mutations. We had chosen this region in particular because of its postulated importance for prion conversion. In addition, dominant negative inhibition of ΔPrPs with deletion of the B2 region has been previously reported [27] and we could expect that some of our mutant PrPs with deletions near B2 would show efficient DNI. If the DNI is then attenuated at a certain point when the deletion is C-terminally extended, this transition would be suggestive of the region involved in PrP-PrP interaction. For convenience of detection, all ΔPrPs contain a 3F4-epitope tag. First, we studied the expression of ΔPrPs upon transient transfection into murine neuroblastoma (N2a) cells. All constructs were expressed at comparable levels (**Fig. 1B**, lanes 2–10), although substantially lower than a 3F4-tagged wild-type PrP [(3F4)MoPrP] used as control (**Fig. 1B**, lane 1). Immunoblot appearance of ΔPrPs were also uniform with more demarcated and narrower bands than that of (3F4)MoPrP and with the diglycoform (**Fig. 1B**, closed arrowhead) predominant over the mono- and non-glycoforms (**Fig. 1B**, open arrowheads). Interestingly, Δ159–167 provided another band over the presumed diglycoform band (**Fig. 1B**, lane 7 and arrow). This was proven to be an extra N-glycan attached due to creation of a third glycosylation sequon (Asn-Tyr-Ser169) by this deletion. Digestion with endoglycosidase H (EndoH) resulted in the expected non-glycoform band (**Fig. 2B**, lane 3, arrowhead). Further proof for this was obtained when serine 169 was deleted [Δ159–167(169)], resulting in the previous glycosylation pattern (**Fig. 2B**, lane 2 and 4).

Taken together, all mutant PrPs were expressed in neuronal cells at comparable levels and with similar immunoblot patterns.

### Confirmation of the dominant negative inhibitory effect of mutant PrPs

For evaluating the DNI of ΔPrPs, we co-transfected ΔPrPs with (3F4)MoPrP in a 1∶1 ratio and evaluated DNI on the co-transfected (3F4)MoPrP, rather than on endogenous PrP^Sc^. As shown previously [17], this method is very sensitive in detecting differences in newly formed PrP^Sc^ levels, requires shorter transfection times and thereby minimizes possible side effects caused by aberrant properties of ΔPrPs. We initially engineered the two constructs with the shortest and longest deletions, Δ159 and Δ159–175, and went on to confirm their DNI. We co-transfected them with (3F4)MoPrP into N2a cells persistently infected with 22L prions (22L-ScN2a) or transfected them alone (**Fig. 1C**). Cells were lysed, lysates were subjected to proteinase K (PK) digestion, separated by SDS-PAGE and evaluated for PrP^Sc^ levels in immunoblot analysis using the 3F4 monoclonal antibody (mAb). Under conditions as used for PK digestion (25 µg/ml for 30 min), ΔPrPs expressed in 22L-ScN2a cells were completely digested (**Fig. 1C**, lanes 5 and 6; **Fig. S1**) and 3F4-immunopositive PrP^Sc^ in co-transfection represents only those derived from (3F4)MoPrP (**Fig. 1D**). When performing co-transfections there were significant differences between the two constructs. Δ159 reduced PrP^Sc^ levels of (3F4)MoPrP to 20% of the empty-vector control (**Fig. 1D**, lane 1 and 2), whereas Δ159–175 reduced only to ∼70% (**Fig. 1D**, representative immunoblot and densitometric analysis).

Taken together, we show that Δ159 and Δ159–175 are not converted into PrP^Sc^ but are able to exert DNI on co-transfected wild-type PrP. Interestingly, Δ159 containing only a deletion of a single residue showed a much more pronounced DNI.

### Characterization of biochemical and cellular properties of ΔPrPs

Prion proteins with internal deletions in the H1∼H2 portion had been reported before to be aberrant in that they have EndoH-sensitive high-mannose-type N-glycans, a mainly intracellular localization, detergent insolubility, and protease resistance, all independent of the presence of PrP^Sc^ [27], [37], [38]. As such aberrant properties might affect DNI efficiency and since there was an obvious difference in DNI efficiencies between Δ159 and Δ159–175, we next tested whether there is a detectable difference in biochemical and cellular properties, explaining the difference in DNI between them.

#### ΔPrPs are likely to be retained in the ER

The pattern of ΔPrPs in immunoblot analysis was similar to that of previously reported PrPs with deletions in the H1∼H2 region, which had Endo H-sensitive high-mannose type N-linked glycans and were diffusely localized inside the cells rather than on the cell surface [27], [38]. Those features suggest that they are retained in the ER. We confirmed that ΔPrPs used in our study also have the same type of N-glycans by Endo H deglycosylation, converging them into the non-glycoform band (**Fig. 2A**, left panel, arrowheads), while glycans of endogenous wild-type PrP were hardly affected (**Fig. 2A**, right panel). Similarly, Δ159–167 was converted by Endo H treatment into a non-glycoform band (**Fig. 2B**, lane 3, arrowhead).

Next, we studied the subcellular localization of ΔPrPs by immunofluorescence analysis. As expected, ΔPrPs localized almost completely in intracellular compartments (**Fig. 2C**, right panel, permeabilized cells) and the cell surface was almost immune-negative (**Fig. 2B**, left panel, non-permeabilized cells).

Taken together, Δ159 and Δ159–175 behave very similar and both have Endo H-sensitive high-mannose type N-linked glycans and are localized intracellularly. These features are highly reminiscent of PrPs retained in the ER.

#### ΔPrPs spontaneously form aggregates

**Figure 3:**
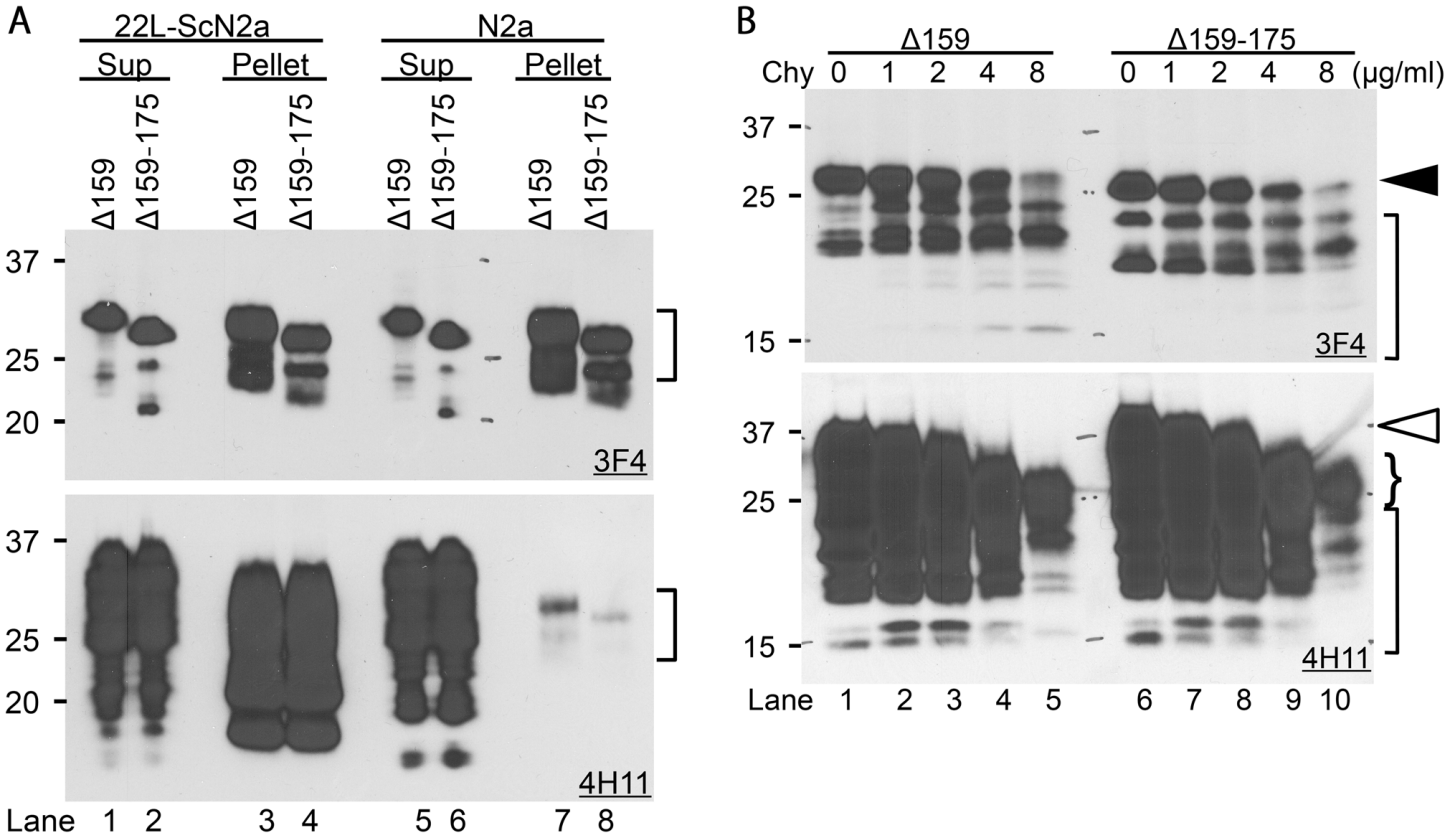
ΔPrPs spontaneously form aggregates that are detergent-insoluble and relatively chymotrypsin-resistant. **A.** ΔPrPs are insoluble in detergent solution. Immunoblots showing partition of Δ159 and Δ159–175 in supernatant (Sup) and pellet fraction after ultracentrifugation of lysates supplemented with 4% sarcosyl. Upper panel is mAb 3F4, lower panel is membrane re-probed with mAb 4H11 detecting also endogenous wild-type PrP besides ΔPrPs. The majority of ΔPrPs is found in the insoluble fraction in both infected and non–infected cells (upper panel, lanes 3, 4, 7 and 8). Wild-type PrP almost completely partitions into the soluble fraction (Sup) in non-infected N2a cells with present conditions. PrP in pellet fractions of non-infected N2a cells is from ΔPrPs as can be seen from the banding pattern (Lane 7 and 8, square bracket). PrP in lanes 3 and 4 (lower panel) is PrP27-30. **B.** ΔPrPs are moderately resistant to chymotrypsin digestion. Immunoblots showing chymotrypsin-resistant fragments of Δ159 and Δ159–175 from transiently transfected N2a cell lysates digested with indicated concentrations of chymotrypsin (Chy). Note that full-length diglycoform of ΔPrP still remains at 8 µg/ml Chy (lanes 5 and 10, upper panel, closed arrowhead), whereas that of endogenous wild-type PrP is completely digested (lanes 5 and 10, lower panel, open arrowhead) and only small amounts of truncated fragments remained (curly bracket). Other smaller fragments in lane 5 and 10 (square-brackets) represent ΔPrPs.

Internally-deleted PrPs, e.g. MHM2PrP(Δ23–88, Δ141–176) (often referred to as PrP106), have been reported to form oligomers and β-sheet structures, becoming protease-resistant and detergent-insoluble [39]–[41]. Likewise, our ΔPrPs were rather insoluble even in 4% sarcosyl solution and largely were found in the pellet fraction, whereas most of endogenous wild-type PrP^c^ was completely solubilized (**Fig. 3A**, lanes 5–8, upper panel transfected PrP (mAb 3F4), lower panel total PrP (mAb 4H11)). Of note, there was no difference between ΔPrPs from 22L-infected and non-infected cells (**Fig. 3A**, lane 3 vs. 7, 4 vs. 8), demonstrating that their detergent insolubility is independent of and unaffected by PrP^Sc^. It was also found that ΔPrPs were more resistant to chymotrypsin digestion than endogenous wild-type PrP. Substantial amounts of full-length ΔPrPs (**Fig. 3B**, upper panel, closed arrowhead) were still remaining even at 8 µg/ml treatment (**Fig. 3B**, lanes 5 and 10), at which full-length endogenous PrP (**Fig. 3B**, lower panel, open arrowhead) was completely digested and only truncated fragments were present at low amounts (curly bracket).

In summary, ΔPrPs are spontaneously forming aggregates which are mildly chymotrypsin-resistant. There was no detectable difference between Δ159 and Δ159–175 in these properties.

#### ΔPrPs are GPI-anchored

**Figure 4:**
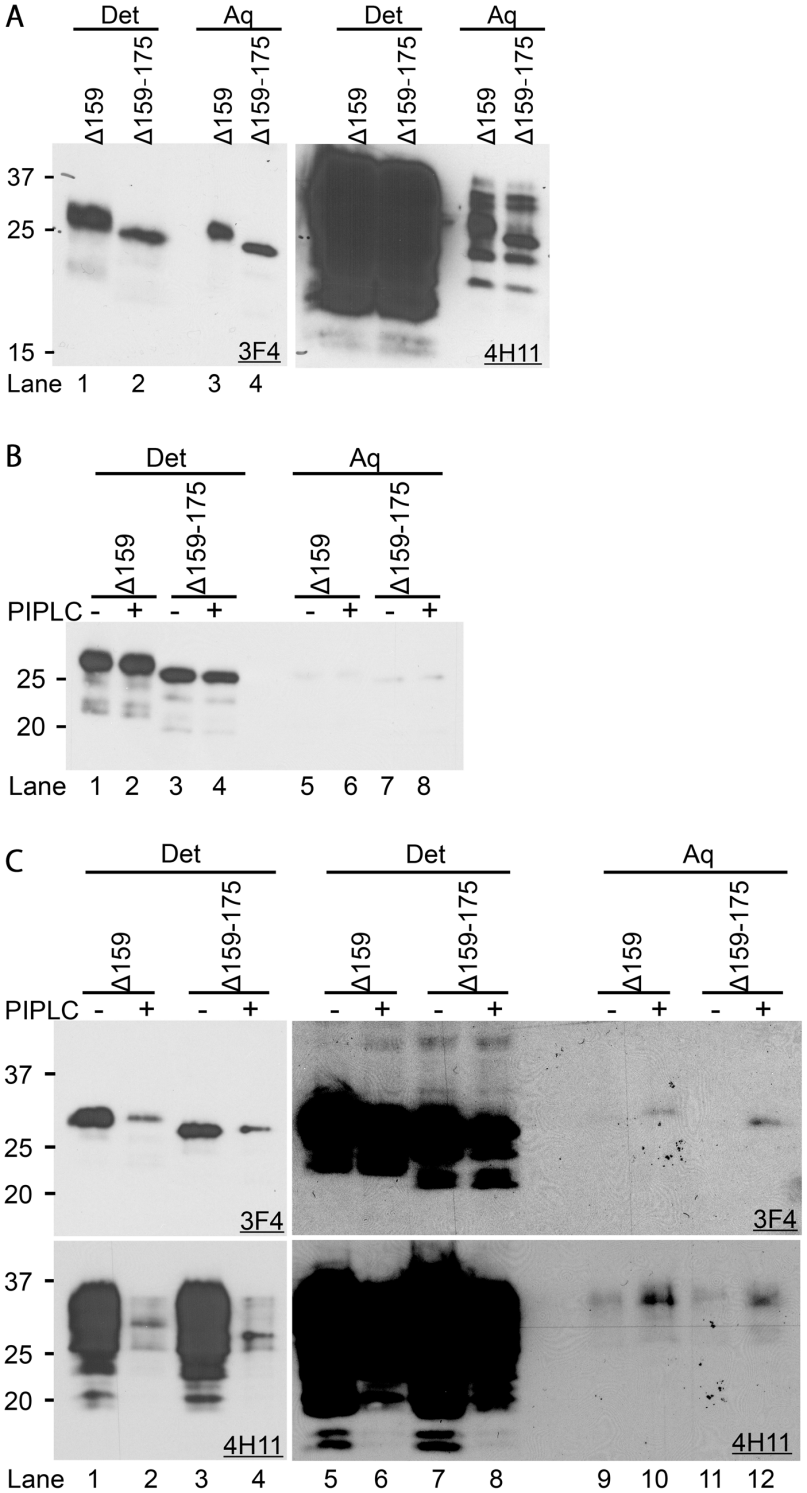
ΔPrPs have a GPI-anchor attached. **A.** ΔPrPs are substantially soluble in detergent without GdnHCl denaturation. Immunoblots probed with mAbs 3F4 (left) or 4H11 (right, membrane re-probed) showing ΔPrPs and endogenous PrP in detergent and aqueous phases of TX114 lysates prepared from N2a cells transfected with Δ159 or Δ159–175. Det denotes detergent phase, Aq aqueous phase. **B.** ΔPrPs are highly hydrophobic after denaturation with GdnHCl. Representative immunoblot of TX114-treated lysates after denaturation with 3 M GdnHCl and developed with mAb 3F4 showing Δ159 and Δ159–175 almost exclusively in detergent phase (lanes 1–4). PIPLC treatment (+, even lanes) was done after denaturation with GdnHCl or not (−, uneven lanes). PIPLC treatment did not significantly influence ΔPrP partitioning under these conditions. **C.** Hydrophobicity of ΔPrPs combined with sensitivity to PIPLC digestion. After further diluting out GdnHCl by phase-separation and removal of the aqueous phase containing GdnHCl, PIPLC was able to digest ΔPrPs. Left two panels (upper mAb 3F4, lower 4H11) are shorter expositions, right panels longer expositions to show also difference in PrP levels in the detergent phase. Under these conditions a substantial reduction of ΔPrPs in the detergent phase was achieved by PIPLC digestion (e.g. upper-left panel, lanes 1, 3 vs. 2, 4). Migration of ΔPrPs in the aqueous phase of PIPLC-digested samples was slightly slower than that of PIPLC-undigested samples (−) (upper-right panel, lane 9 vs. 10).

So far glycosylphosphatidylinositol (GPI) anchoring of internally-deleted PrPs was controversial. It was reported that PrP106 has a GPI anchor [37], whereas other internally-deleted PrPs were reported to lack it [38]. As GPI anchoring greatly affects properties of PrP, especially intracellular trafficking and interaction with PrP^Sc^ [42], [43], we wanted to solve this issue for our mutant PrPs using Triton X-114 (TX114) extraction. First, we phase-separated TX114 lysates of cells expressing Δ159 and Δ159–175, respectively, and evaluated partition in detergent and aqueous phase. Although more than half of ΔPrPs was sequestered to the detergent phase, substantial amounts remained in the aqueous phase (**Fig. 4A**, left panel). In contrast, the vast majority of endogenous wild-type PrP was extracted to the detergent phase (**Fig. 4A**, right panel). As such a pattern of separation can be also seen with transmembrane proteins, e.g. lysosome-associated membrane protein-1 (LAMP1) used here as a control (*data not shown*), adequate GPI anchoring was still questionable at this point.

Since we considered that the aggregate nature of ΔPrP might conceal their hydrophobic parts, including the lipid moiety of the GPI-anchor, we tried GdnHCl treatment of ΔPrPs before phase separation to disintegrate the aggregates. As expected, the majority of ΔPrPs were now extracted to the detergent phase (**Fig. 4B**, lanes 1 vs. 5, 3 vs. 7). To further confirm GPI-anchoring, *in vitro* digestion with PIPLC was done before phase separation. Although we diluted out GdnHCl to ∼0.5 M, there was no difference between samples with and without PIPLC treatment (**Fig. 4B**, lane 1 vs. 2, 3 vs. 4), presumably because GdnHCl concentration was still too high for PIPLC digestion. We further diluted GdnHCl by removing the aqueous phase after the first phase separation and diluted the detergent phase with fresh buffer. Eventually, GdnHCl could be lowered to sufficient levels that PIPLC was able to digest ΔPrPs and endogenous PrP (**Fig. 4C**, upper panel mAb 3F4, lower panel mAb 4H11 for total PrP). There was a clear reduction of ΔPrP levels of lysates digested with PIPLC in the detergent phase along with an increase in the aqueous phase (**Fig. 4C**, lane 9 vs. 10, 11 vs. 12). The same changes were observed for endogenous PrP (**Fig. 4C**, lower panel). The increment of ΔPrPs and endogenous PrP in the aqueous phase of PIPLC-digested samples was much smaller than the decrement in the detergent phase. In addition, PIPLC-digested PrPs migrated slower than undigested ones (**Fig. 4C**, lane 9 vs. 10). These features are consistent with GPI-anchored proteins after PIPLC digestion. Loss of GPI-anchors results in reduction of hydrophobic surfaces, consequently poorer adsorption to PVDF membrane and fewer SDS molecules attached to proteins in SDS-PAGE [44].

Taken together, our findings prove that ΔPrPs are GPI-anchored.

### ΔPrPs are degraded by acidic compartments, presumably involving autophagic processes

**Figure 5:**
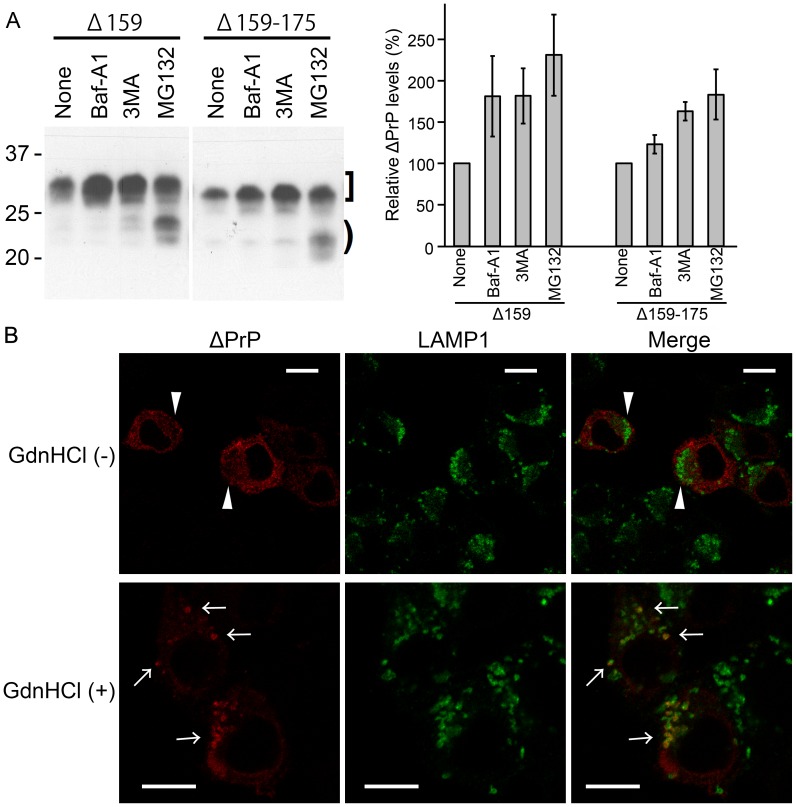
ΔPrPs are degraded by both lysosomal and proteasomal degradation systems. **A.** Degradation of ΔPrPs by lysosomal and proteasomal systems. (Left) N2a cells transiently transfected with Δ159 or Δ159–175 were treated for 7 hours with inhibitors either for lysosomal degradation (bafilomycin A1, Baf, 120 nM), autophagy (3-methyladenine, 3MA, 10 mM), or the proteasome (MG132, 10 µM) and cell lysates analyzed in immunoblot for expression of Δ159 and Δ159–175 (mAb 3F4). Square bracket and bracket denote diglycoforms and nonglycoforms of ΔPrPs, respectively. An increment in diglycoforms is observed in Baf- and 3MA-treated cells, whereas in MG132-treated cells also the non-glycoforms are increased. (Right) A graph showing quantification of all PrP bands by densitometric analysis. Data from 3 independent (one in duplicate) experiments for Δ159 and 2 independent (one in duplicate) for Δ159–175 were statistically analyzed for mean and standard deviation (error bars). **B.** ΔPrPs can be localized in LAMP1-immunopositive vesicles. Immunofluorescence analysis shows distribution of ΔPrP (mAb 3F4) and LAMP1 in cells with or without 6M GdnHCl antigen-retrieval treatment. N2a cells transfected with ΔPrP159 were fixed, permeabilized and incubated without (upper panel) or with 6M GdnHCl (lower panel). With GndHCl treatment ΔPrP is diffusely distributed (arrowheads) and co-localizes poorly with LAMP1. After GdnHCl treatment (lower panel), bright 3F4-immunopositive puncta (arrows) are observed which partly co-localize with LAMP1-positive structures (merge). Scale bars, 10 µm.

Next, we investigated by which degradation systems ΔPrPs are degraded. Since DNI involves a direct physical interaction between PrP^c^ and PrP^Sc^, the subcellular trafficking of ΔPrPs should lead into compartments where PrP^Sc^ molecules reside. First, we studied the effects of inhibitors of lysosomal or proteasomal degradation on levels of ΔPrPs, specifically the V-ATPase inhibitor bafilomycin A1 (Baf), the autophagy inhibitor 3-methyladenine (3MA), and the proteasome inhibitor MG132. Cells were incubated only up to 7 hours to prevent interference with additional degradation systems [45]. There was no difference between Δ159 and Δ159–175, suggesting that these mutant PrPs share the same metabolic pathway. Baf and 3MA most efficiently inhibited degradation of the diglycoform of ΔPrP (**Fig. 5A**, square bracket). Therefore, at least part of ΔPrPs seemed to be transferred to a 3MA-sensitive, presumably class-III PI3K-dependent pathway and eventually degraded in acidic compartments. The banding pattern of ΔPrPs from MG132-treated cells was clearly different from those of Baf or 3MA-treated cells, with a main increase in the non-glycoform of ΔPrP (**Fig. 5A**, bracket), as expected for proteins subjected to endoplasmic reticulum-associated degradation (ERAD) [46].

This data suggests that ΔPrPs undergo a mixed degradation and are subjected both to lysosomal/autophagic pathways and the proteasome.

A lysosomal degradation of ΔPrPs was unexpected, as the EndoH-sensitive N-glycans of ΔPrPs suggested that they would not reach the medial Golgi from which a trafficking pathway to lysosomes has been suggested [47]. To confirm lysosomal degradation, we next studied whether ΔPrPs co-localize with LAMP1, calnexin, Rab7, Rab9 and microtuble-associated protein 1 light chain 3 (LC3) in immunofluorescence analysis/confocal microscopy. Non-transfected cells (for LAMP1 and calnexin) or cells transiently transfected with GFP-Rab7, -Rab9 and -LC3, respectively, were pre-treated with Baf for 6 hours to prevent lysosomal degradation. Without GdnHCl pretreatment of cells we did not observe a significant co-localization of ΔPrPs and LAMP1 (**Fig. 5B**, upper panels, arrowheads), GFP-Rab7 or GFP-Rab9 (*data not shown*). A very weak to moderate co-localization was found for ΔPrPs and LC3 or calnexin, respectively (**Fig. S2** and **S3**). Since ΔPrPs forms aggregates, we hypothesized that the epitope for anti-PrP antibodies might not be accessible, just as is the case for PrP^Sc^ [48]. Therefore, we applied the antigen retrieval procedure for PrP^Sc^ by treating the cells with GdnHCl. After treatment with 6 M GdnHCl, punctuate-vesicular fluorescent structures were observed and some of them clearly co-localized with LAMP1 (**Fig. 5B**, lower panels, arrows). Unfortunately, these harsh conditions were not appropriate for detection of GFP-Rab7, -Rab9 and -LC3 (**Fig. S4**), presumably due to denaturation of EGFP.

To obtain further evidence for lysosomal degradation of ΔPrPs we utilized subcellular fractionation of Δ159 transfected N2a cell lysates on sucrose gradients (**Fig. S5**). Part of the studies involved pre-treatment of cells with bafilomycin A1 to study effects of this drug on distribution of Δ159 in the gradients (**Fig. S5B**). We found that a substantial proportion of Δ159, importantly only the glycosylated form, was present in low-density fractions, between 8.5 and 30% (w/w), where ER components are scarce, whereas late endosomes, lysosomes and autophagosomes are enriched there. This fraction was also most enriched with LAMP1 and LC3-II, and the amounts of the Δ159 diglycoform were strongly increased by bafilomycin A1 treatment (**Fig. S5B**). Calnexin was most abundant in the high-density fraction, specifically in the interphase between 36 and 42% (w/w). Interestingly, the mono- and non-glycoforms of Δ159 were also mainly present in the interphase where calnexin was most enriched. In conclusion, these findings strongly corroborate the microscopic findings that substantial amounts of glycosylated Δ159 are present in amphisomes and lysosomes and are consistent with a lysosomal degradation pathway as suggested by increase of the diglycoforms of ΔPrPs by bafilomycin A1 or 3MA treatment.

Taken together, we demonstrate that a portion of ΔPrPs is subjected to lysosomal degradation by a 3MA-sensitive process.

### DNI of ΔPrPs is inversely correlated with size of deletion

**Figure 6:**
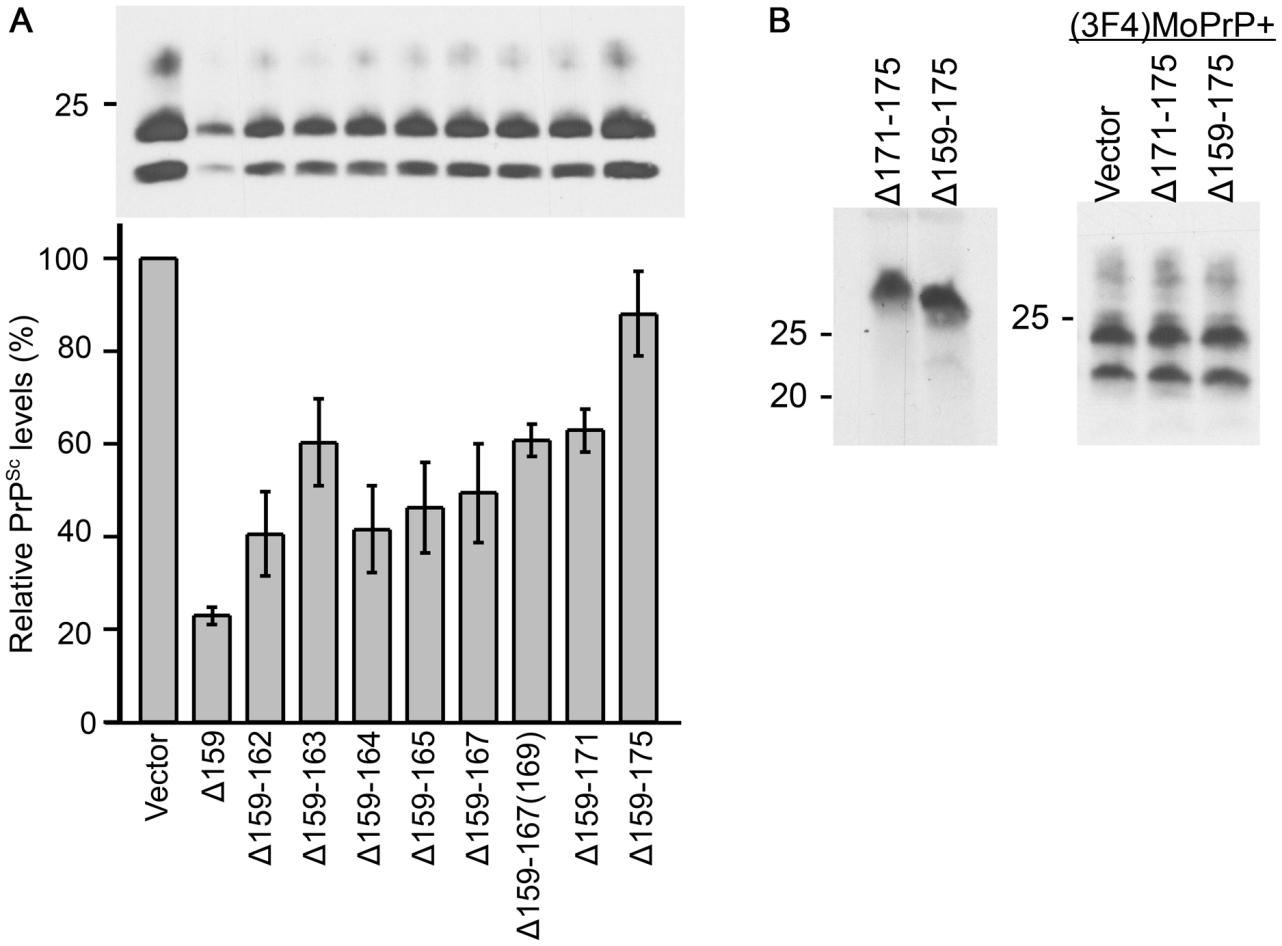
Inverse correlation of DNI and size of deletion and importance of positioning of deletion. **A.** DNI efficiencies of ΔPrPs are inversely correlated to deletion size. Representative immunoblot showing PK-resistant PrP^Sc^ moiety of (3F4)MoPrP co-expressed with indicated ΔPrPs in 22L-ScN2a cells. Quantitative analysis is shown below as scheme. Relative PrP^Sc^ levels were calculated as proportion of PK-resistant (3F4)MoPrP co-transfected with each ΔPrP to that of co-transfected with empty vector control in the same experiment. Data from 4 (for Δ159–165, Δ159–167 and Δ159–167(169) only 3) independent experiments were statistically analyzed for mean and standard deviation (error bars). **B.** DNI of ΔPrP with a deletion in the C-terminal part of H1∼H2. Δ171–175 (see Fig. 7A) was transiently expressed in N2a cells (left panel) and DNI efficiency evaluated in 22L-ScN2a cells (right panel) (mAb 3F4). Despite the much shorter deletion size, Δ171–175 exerted only inefficient DNI, similar to Δ159–175 (right panel).

**Figure 7:**
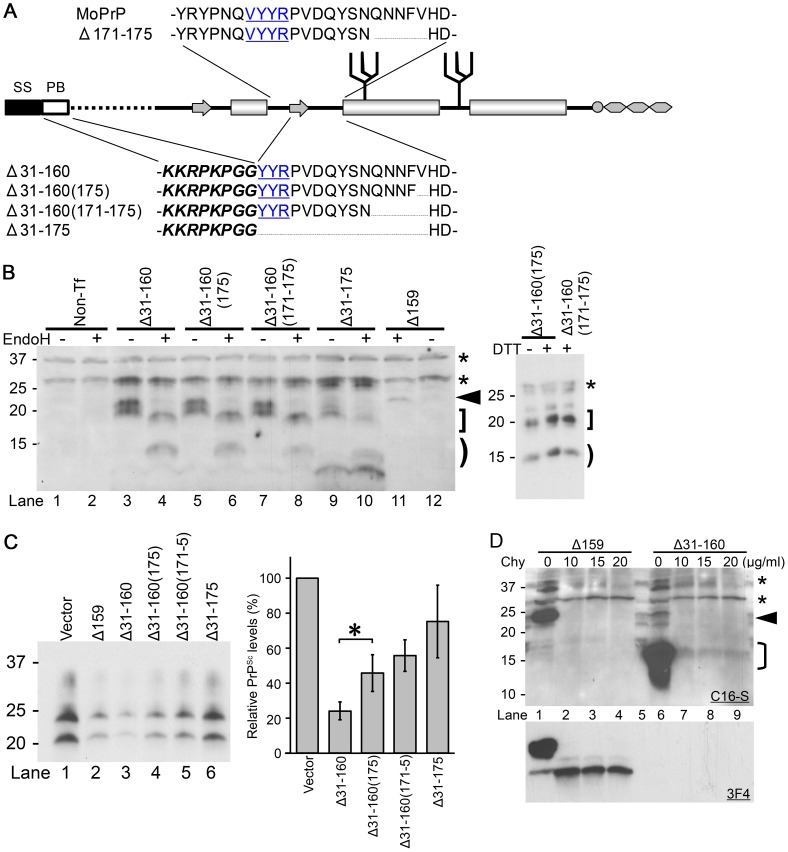
ΔPrPs lacking in addition the N-terminal region still exert efficient DNI. **A.** Schematic illustration of Δ171–175, Δ31–160 and its variants. Δ31–160 variants lack the entire part between the polybasic motif (PB, italic and bold) and residue 161. SS, signal sequence. **B.** Δ31–160 variants are expressed at comparable levels and have EndoH-sensitive and –resistant N-glycans. Immunoblot comparing EndoH-digested and non-digested samples from N2a cells transiently transfected with Δ31–160 variants, probed with anti-PrP mAb C16-S raised against the C-terminal portion of H3. Non-Tf, samples prepared from N2a cells without transfection. Arrowhead denotes deglycosylated Δ159 (lane 11), bracket deglycosylated fragments, square bracket EndoH-resistant fragments. Asterisks indicate non-specific bands. Δ31–160 variants are not completely deglycosylated by EndoH, resulting in EndoH-resistant fragments (square bracket) which are observed also in reducing conditions (right panel; +/− dithiothreitol (DTT) treatment). Note that endogenous wild-type PrP^c^ was not detected under used conditions. **C.** DNI of Δ31–160 variants is similarly inversely related to size of deletion and dependent on intact C-terminal H1–H2 portion. Immunoblot probed with mAb 3F4 showing PK-resistant (3F4)MoPrP co-transfected with indicated constructs into 22L-ScN2a cells (left panel). Δ31–160 was slightly more effective than Δ159. Right panel shows quantification of results as obtained from a triplicate experiment, using densitometry on ImageJ. Bars illustrate mean ± standard deviation. *, p<0.05. **D.** Δ31–160 forms spontaneous aggregates with moderate chymotrypsin (Chy) resistance. Immunoblots probed with mAbs C16-S (upper panel) or 3F4 (lower panel) for comparing Chy-resistant fragments of Δ31–160 with that of Δ159. Lysates from N2a cells expressing Δ159 or Δ31–160 were digested with indicated concentrations of Chy for 30 minutes and then digested with PNGaseF. A sample from non-transfected N2a cells is shown in the lane 5. Asterisks indicate non-specific C16-S bands. Arrowhead denotes deglycoform of Δ159, bracket deglycoform of Δ31–160.

As there was no detectable difference in biochemical or cell-biological properties between Δ159 and Δ159–175, we speculated that their difference in DNI is most likely attributable to the size and positioning of the internal deletions. Therefore, we next studied DNI efficiencies of all ΔPrPs by co-transfection with (3F4)MoPrP (**Fig. 6A**). DNI efficiencies of Δ159 and Δ159–175 were reproduced with PrP^Sc^ levels ∼20% and 70–80%, respectively, and ΔPrPs with deletions between these two constructs showed a gradual reduction of DNI efficiency as deletions were extended, i.e. an inverse relation between DNI efficiency and deletion size (**Fig. 6A**). The relatively low DNI efficiency of Δ159–163 might be due to the unique primary structure with two prolines and only one intervening residue, making the regional structure less flexible and disadvantageous for inter-PrP interaction (**Fig. 1A**). On the other hand, Δ159–167 showed a comparable DNI effect to adjacent ΔPrPs, despite the extra N-glycan (**Fig. 1B**). Next, we assessed whether small deletions in the C-terminal part of H1-H2 exert DNI. PrPΔ171–175 (**Fig. 7A**) was expressed at similar levels as Δ159–175 (**Fig. 6B**, left panel) and, interestingly, its DNI efficiency was also comparable to that of Δ159–175 (**Fig. 6B**, right panel), suggesting that the length of H1∼H2 is not the sole determinant of DNI and that the positioning of the mutation is also important for efficient DNI. Taken together, all PrPs with deletions in the H1–H2 region exerted DNI, although more pronounced when the C-terminal part was preserved.

### The region N-terminal to H1∼H2 is not necessary for efficient DNI

The observed correlation between DNI efficiencies and the size and/or positioning of the internal deletion suggested that the H1∼H2 region is critically important for PrP-PrP interaction, presumably forming part of the interaction interface. However, there was still the possibility that ΔPrPs with smaller deletions had higher affinities, hence more efficient DNI, because their entire molecular structure was more similar to intact PrP^c^, whereas those with larger deletions had lower affinities. Alternatively, the interaction interface might be a discontinuous epitope over N- and C-terminal domains with H1∼H2 being the hinge connecting the domains. In such scenarios H1∼H2 itself would not contribute to the interaction interface. To address this experimentally, we created ΔPrPs lacking the entire sequence from residue 31 to 160 (Δ31–160) and tested their DNI. We preserved the polybasic motif (residues 23–28) and the following glycine residues (**Fig. 7A**), because this sequence was reported to affect subcellular trafficking and DNI [47], [49]. In addition, we created variants of Δ31–160 with deletions in the C-terminal end of H1∼H2, namely Δ31–160(175), Δ31–160(171–175), and Δ31–175 (**Fig. 7A**). All constructs were expressed at similar levels upon transient transfection and banding patterns were similar before and when subjected to EndoH digestion, suggesting a similar subcellular trafficking and metabolism. Of note, their N-glycans were not completely EndoH-sensitive with an additional band (**Fig. 7B**, left panel, square bracket) not converged to the non-glycoform band (**Fig. 7B**, left panel, bracket). This feature is different from that of ΔPrPs presented above. These additional bands were even unchanged after treatment with 100mM of DTT (**Fig. 7B**, right panel), although they were absent in PNGaseF treated samples (**Fig. 7D**, lane 6). In summary, it was evident that a substantial part of N-glycans of Δ31–160 and variants of it were EndoH-resistant. This fraction of PrP might exit ER, pass medial Golgi and reach endosomal/lysosomal compartments [47] or the cell surface, being consistent with the cell-surface distribution of a mutant PrP with the similar structure [50].

Again, all constructs were expressed at similar levels and their efficiencies varied substantially (**Fig. 7C**, right panel). Δ31–160 showed efficient DNI, whereas DNI of Δ31–160(175) was significantly less, although only one additional residue was deleted (**Fig. 7C**). As before, there was an inverse correlation between size of deletion and DNI. DNI of Δ31–175 which almost completely lacks H1∼H2 was rather low as expected (**Fig. 7C**, lane 6). Compared to ΔPrPs with preserved N-terminal portions, PrPs lacking the N-terminal region were higher expressed (see **Fig. 7B**, lane 11, arrowhead), which might bias their DNI efficacy. We finally analyzed protease resistance of these constructs and found that they were mildly resistant to chymotrypsin digestion (**Fig. 7D**).

Taken together, ΔPrPs lacking the N-terminal part of PrP were higher expressed and showed a different EndoH resistance pattern. The N-terminal part of PrP was not necessary for exerting DNI and the inverse relationship between size of deletion within the H1∼H2 region and DNI was preserved.

## Discussion

It is well accepted that the PrP^C^-PrP^Sc^ conversion process consists of an initial binding step and subsequent refolding events to generate authentic PrP^Sc^ [17], [51]–[53]. In the present study, we focused on investigation of the binding step in prion-infected cell culture models. To specifically and systematically analyze binding efficiencies in living cells, we used a series of internally deleted prion proteins (ΔPrPs) whose prion conversion capabilities were deliberately ablated, in conjunction with co-transfected wild-type PrP. Although predictable aberrant biochemical properties of ΔPrPs initially posed concerns, eventually ΔPrPs were uniformly expressed and this feature did not interfere with evaluation of dominant negative effects on prion conversion (DNI). On the contrary, those aberrant properties of ΔPrPs revealed novel insights into the cell biology of prion proteins.

### Underlying mechanism of DNI exerted by ΔPrPs

A question of superior importance in the interpretation of our results is what the interaction target of ΔPrPs is to exert their DNI. Initially, protein or factor X has been postulated as target of mutant PrPs for exerting DNI [17]. Recent studies using in vitro conversion systems, e.g. protein misfolding chain amplification (PMCA), have denied the necessity for other factors and DNI in vitro can occur only between the substrate PrP, template PrP^Sc^, and the inhibitory mutant PrPs [25], [28]. From a stoichiometric point of view, an interaction between PrP^Sc^ and inhibitory mutant PrPs was the most likely cause of DNI [25]. These in vitro findings in concert with missing experimental evidence for a factor X make it reasonable to consider that ΔPrPs exert DNI in prion-infected cultured cells by direct interaction with template PrP^Sc^ or substrate PrP^C^. Of importance is also the vast difference in expression levels when substrate (3F4)MoPrP and ΔPrPs are co-transfected. Although ΔPrPs are much less expressed, they are nevertheless able to exert substantial DNI effects, as was also reported before [27]. This suggested that the interaction target of ΔPrPs is PrP^Sc^ and not PrP^C^. However, the possibility that substrate PrP^C^ is a target still cannot be fully excluded, given that only a small moiety of the total PrP^C^ population might find its way into the cellular compartment of prion conversion, where ΔPrPs also have access as we report here.

The variation in DNI efficiencies between Δ159, Δ171–175 and Δ159–175 suggests a critical importance of the integrity of the H1∼H2 region and its positioning relative to helix 2 and 3. We did not expect to see a similar tendency in DNI variation among the group of ΔPrPs which contain deletions of almost the entire stretch N-terminal to H1∼H2, like Δ31–160, Δ31–160(175), Δ31–160(171–175) and Δ31–175. Unfortunately, we cannot directly compare DNI efficiencies of Δ31–160 and Δ159 to deduce the direct contribution of the N-terminal deleted region in DNI, e.g. by calculating a ‘DNI per PrP mutant’ of each. This is because their properties seem to be rather distinct from each other, especially with regard to EndoH sensitivity which reflects subcellular localization and trafficking. In addition, Δ31–160 lacks almost the entire N-terminal region, including pre-octarepeat and octarepeat regions, deficiencies of which were shown to impair interaction efficiencies of mutant PrPs with PrP^Sc^ and/or PrP^C^ [54]–[56]. However, the obviously very efficient DNI of Δ31–160 demonstrates that the DNI contribution of the region encompassing residue 32 to the end of H1 is very small to negligible in our experimental set-up.

In any case, the finding that Δ31–160 variants with additional deletions in H1∼H2 behaved very similar in DNI as the initial ΔPrPs strongly corroborates our findings and its interpretation. Among the currently postulated models for PrP^Sc^, our results are best compatible with the “domain-swapping model” or the “parallel in-register extended β-sheet model” [32], [57]–[59]. Here, H1∼H2 forms intermolecular anti-parallel or parallel β-sheets and H2 and H3 contribute to the stabilization of the superstructure by interaction with H1∼H2, H2 and/or H3 of the other PrP molecule [32], [60]. With such models, the inverse correlation between deletion size and DNI efficiency of ΔPrPs can be explained by changes in the surface area of the interaction interface. In addition, the requirement for appropriate positioning of H1∼H2 relative to H2 and H3 is also explained. Interestingly, these models with H1∼H2 forming intermolecular β-sheet structures were mainly based on in vitro synthesized PrP fibrils [32], [57], [60]. So it is conceivable that the modalities of PrP-PrP interaction as postulated in fibril formation in vitro are relevant also in living cells and form the molecular basis for DNI as observed here. In case full-length PrP and ΔPrPs share similar DNI mechanisms, the implications from the present study might be more widely applicable to scenarios which involve full-length PrP molecules.

There is a caveat to this point of view. It is the transgenic mice expressing PrP106, which completely lack the H1∼H2 region and nevertheless develop prion disease following inoculation with scrapie prions, albeit with extended incubation periods [37]. This suggests that PrP-PrP interactions still occur in this case. On the other hand, PrP106 did not show any DNI in transgenic mice co-expressing endogenous PrP and PrP106 [37], which is again consistent with our hypothesis. One explanation is that PrP106 still maintains some affinity for PrP^Sc^ through other regions than H1∼H2. This point of view is supported by our findings that even Δ159–175 and Δ31–175 still maintain a certain degree of DNI, although much less pronounced. In addition, H2 and H3 have been reported to have a substantial aggregation tendency to form fibrils in vitro [61]. Such weak affinity might be sufficient for PrP106 to facilitate prion propagation in vivo. Once PrP106 has acquired a PrP^Sc^ conformation, such nascent PrP^Sc^ composed of PrP106 would convert normal-isoform PrP106 much more efficiently than would do wild-type PrP^Sc^.

The B2-H2 loop is thought to be important for prion propagation especially in inter-species transmission situations [31]. Interestingly, our data suggest that H1∼H2 including the B2-H2 loop is a critical determinant of DNI efficiencies. If full-length PrP interacts with PrP^C^ or PrP^Sc^ in the same modalities as does ΔPrP, H1∼H2, along with the B2-H2 loop, might be important for prion propagation because it affects PrP-PrP-interaction efficiencies. Investigations to test whether ΔPrPs and full-length PrPs similarly interact with PrP^Sc^ are under way. Further investigation of ΔPrP effects would also have practical implications. Identification of the structure which is required for efficient PrP-PrP binding can provide novel therapeutic targets and might lead to the development of small-molecule compounds which recognize PrP structural elements and hamper PrP-PrP interaction.

### ΔPrPs have access to locales of prion conversion although not reaching the mid-Golgi: a new trafficking route for prion proteins?

For exerting DNI which involves a direct physical interaction between ΔPrP and PrP^Sc^, these two proteins have to meet, ideally in the cellular locale of prion conversion. On the other hand, ΔPrPs were not complex glycosylated and were obviously subject to the known cellular quality control mechanisms in the secretory pathway. They did not reach the plasma membrane and were retained in ER and early-Golgi compartments. To resolve this obvious paradox, another focus of our analysis was on the subcellular trafficking and degradation pathways of these prion proteins. This lead to the finding that ΔPrPs undergo a mixed degradation and have access to subcellular trafficking routes previously not assumed for prion proteins.

A first unexpected finding was that the ΔPrPs studied here have GPI anchors attached, which is of importance as it affects subcellular trafficking and metabolism. An even more surprising finding was that ΔPrPs are subject to both the lysosomal and proteasomal degradation system. The effects of proteasome inhibitors on ΔPrP levels were also unexpected as GPI-anchored PrPs have previously been reported to be unsuitable for ERAD and proteasomal degradation [46]. The lysosomal degradation of ΔPrPs is even more interesting because it explains how ΔPrPs can eventually encounter template PrP^Sc^. We and others have previously reported a post-ER cellular quality control pathway which re-routes aggregated PrP or EndoH-resistant mutant PrPs from Golgi apparatus or TGN to acidic digestive compartments [47], [62]. Unlike such EndoH-resistant mutant PrPs which reach at least the medial or late Golgi compartment, ΔPrPs are obviously retained earlier and their trafficking pathway to lysosomes must be a different one. The pronounced sensitivity to 3MA inhibition suggests that this process is class III PI_3_K-dependent and part of the macro-autophagy pathway. A similar degradation mechanism for ER-retained proteins, e.g. glycoproteins with high-mannose-type N-glycans [63], misfolded dysferlin [64] and procollagen aggregates [65], was already reported. It utilizes autophagic sequestration and eventually directs these proteins in autophagic vesicles to lysosomes. The same system might be operative in the lysosomal degradation of ΔPrPs. Importantly, before fusion with lysosomes and degradation of its contents, autophagosomes can fuse with late endosomes to form amphisomes which are immunopositive both for LC3 and LAMP1 [66]. Possibly, such amphisomes are the site where ΔPrPs encounter PrP^Sc^ template first. Of note, this observation might provide novel insights into the cellular biology of prion conversion and involved trafficking and re-cycling pathways. It is clear that substrate PrP^C^ is converted to PrP^Sc^ either at the plasma membrane [67] or after endocytosis on the way to lysosomes where it undergoes N-terminal truncation [9], [68], [69]. There is now good experimental evidence that a main intracellular locale of prion conversion is the ERC compartment [70], [71] which strongly implies that there is a re-cycling of PrP^Sc^ back towards the plasma membrane, in order to sustain continuous presence of PrP^Sc^ template in conversion-competent compartments. Of note, there is no classical trafficking pathway described from late endosomes back to ERC or early endosomes. On the other hand, a re-cycling of PrP^Sc^ molecules from late endosomes to TGN has been reported by us and others [35], [72]. From there, such re-routed PrP^Sc^ has access to either ERC or plasma membrane, closing the cycle.

In the context of our experimental findings, ΔPrPs re-routed to late endosomes via autophagy pathways have to reach template PrP^Sc^ or substrate PrP^c^ in a stage before conversion into *bona fide* PrP^Sc^ is accomplished. Whether this is the case in late endosomes fused with amphisomes is questionable, although not impossible. It is conceivable that the process of making bona fide and fully PK resistant PrP^Sc^ is a multi-step pathway which involves more than one cellular compartment. In addition, also ΔPrPs might be subjected to the above described re-cycling from late endosomes/amphisomes back to TGN and plasma membrane. Interestingly, the stoichiometry is not in favor of ΔPrPs compared to wild-type PrP, nevertheless they exert a very efficient DNI. Either only a certain minor subpopulation of PrP^c^ is prone to be converted into PrP^Sc^ or ΔPrPs have access to a locale which is extremely powerful in the process of cellular prion conversion.

Another implication of our work is that other proteins or factors residing in ER and early Golgi might also have access to the locale of prion conversion. Without using the known exocytic and endocytic pathways such factors could be involved in the course of prion conversion or PrP^Sc^ degradation. Given such a scenario, even ER-resident proteins, e.g. ER chaperons, might have the possibility to interact with substrate PrP^C^ or template PrP^Sc^. Alternatively, the non-protein co-factors as mainly described in in vitro systems [73]–[76] might get access. The confinement of such co-factors in small vesicles might change their stoichiometry and their ability to negatively or positively interfere with prion conversion and propagation.

Overall, our data reinforce the notion that autophagy pathways can influence prion propagation [77]. We also show that the intracellular trafficking of PrP isoforms is much more complex than previously anticipated.

## Supporting Information

Figure S1 **ΔPrPs are completely digested by PK at 25 µg/ml.** Representative immunoblot probed with mAb 3F4 showing samples from 22L-ScN2a cells transiently transfected solely with plasmids encoding indicated ΔPrPs (even lanes) or co-transfected with mixture of same amounts of plasmids encoding respective ΔPrP and (3F4)MoPrP (odd lanes). Cells were harvested 24 hours after transfection and lysates digested with PK at 25 µg/ml for 30 minutes. Note that ΔPrPs were completely digested when transfected alone, proving that PK-resistant PrP detected in co-transfected cells represents solely those of (3F4)MoPrP.
(TIF)

Figure S2 **ΔPrP is moderately co-localized with calnexin (CNX).** Confocal microscopy analysis of N2a cells transiently transfected with Δ159 and immuno-labeled with mAb 3F4 and anti-CNX polyclonal antibody. A section from the level of nuclei is shown. Note that both ΔPrP and CNX are diffusely distributed inside cells and show only a moderate degree of co-localization. Scale bar, 25 µm.
(TIF)

Figure S3 **ΔPrP poorly co-localizes with EGFP-LC3 puncta.** Confocal microscopy analysis of N2a cells transiently co-transfected with Δ159 and EGFP-LC3 and immuno-labeled with mAb 3F4. A section from the level of nuclei is shown, without GdnHCl treatment. Auto-fluorescent EGFP-LC3 puncta do not well co-localize with intensive fluorescent ΔPrP spots. Of note, this represents levels of basal autophagy without any induction of autophagy. Scale bar, 10 µm.
(TIF)

Figure S4 **Attempts to observe co-localization of Δ159 with GFP-Rab7 or -Rab9.** Confocal microscopy analysis of N2a cells co-transfected with Δ159 and EGFP-Rab7 or EGFP-Rab9. Transiently transfected N2a cells were fixed on cover slips and incubated with the indicated concentrations of GdnHCl for 45 minutes, followed by immuno-labeling with mAb 3F4 and DyLight594-conjugated anti-mouse IgG antibody. A section at the level of nuclei was used for co-localization analysis. EGFP-Rab7 and -Rab9 are found as blurry spots after GdnHCl treatment and a significant co-localization with 3F4-immunopositive structures is not found. Scale bars, 10 µm.
(TIF)

Figure S5 **Subcellular fractionation provides further evidence for lysosomal degradation of Δ159.** **A.** Subcellular fractionation on sucrose gradients consisting of 8.5%, 33% and 36% overlaid on homogenate (with 42% sucrose) from N2a cells with or without transfection of Δ159 (Non-Tf, non-transfected cells). A substantial amount of Δ159 is distributed to the 8.5%/33% interphase where calnexin (CNX) is relatively scarce (lower panel). Note that most of Δ159 in the 8.5%/33% interphase is diglycoform, while mono- and non-glycoforms are mainly present in the 36%/42% interphase where CNX is most enriched. **B.** Subcellular fractionation on sucrose gradients consisting of 8.5%, 30% and 36% overlaid on homogenate (with 42% sucrose) from transfected N2a cells with (Baf +) or without (Baf −) treatment with bafilomycin A1. Substantial amounts of Δ159 are reproducibly distributed to the low-density fraction (8.5%/30% interphase), where CNX is scarce and LAMP1 and LC3-II are most enriched. Amounts of the Δ159 diglycoform were strongly increased by bafilomycin A1 treatment. On the right a longer exposition is shown for PrP and LC3. The blot was simultaneously developed for PrP, LC3 and LAMP1, and then re-probed with anti-CNX antibody (αCNX). WCL, whole-cell lysates without fractionation.
(TIF)

Table S1 **Primers used for creating ΔPrPs.** The term “Rev” in primer names indicates anti-sense primers. Antisense primer “Common Rev. Δ159-X” was combined with sense primers whose names start with “Δ159-” to create the internal deletions as indicated by the name of the sense primers by site-directed mutagenesis. Likewise, primer “Common ΔX-175” was combined with antisense primers whose names end with “-175” to create the internal deletions as indicated by their names. Δ31–160 was created by combining primers “ΔX-160” and “Rev. Δ31–160”. Δ171–175 was created by using primers “Δ171–175” and “RevΔ171–175”. Δ159–167(169) was accidentally created when engineering Δ159–167.
(PDF)

## References

[1] Prusiner SB (1998) Prions. Proc Natl Acad Sci U S A 95: 13363–13383. 9811807 10.1073/pnas.95.23.13363 PMC33918

[2] Weissmann C, Li J, Mahal SP, Browning S (2011) Prions on the move. EMBO Rep 12: 1109–1117. 21997298 10.1038/embor.2011.192 PMC3207107

[3] Aguzzi A, Polymenidou M (2004) Mammalian prion biology: one century of evolving concepts. Cell 116: 313–327. 14744440 10.1016/s0092-8674(03)01031-6

[4] Collinge J (2001) Prion diseases of humans and animals: their causes and molecular basis. Annu Rev Neurosci 24: 519–550. 11283320 10.1146/annurev.neuro.24.1.519

[5] Watts JC, Balachandran A, Westaway D (2006) The expanding universe of prion diseases. PLoS Pathog 2: e26. 16609731 10.1371/journal.ppat.0020026 PMC1434791

[6] Prusiner SB (1982) Novel proteinaceous infectious particles cause scrapie. Science 216: 136–144. 6801762 10.1126/science.6801762

[7] Cohen FE, Pan K-M, Huang Z, Baldwin M, Fletterick RJ, et al (1994) Structural clues to prion replication. Science 264: 530–531. 7909169 10.1126/science.7909169

[8] Borchelt DR, Scott M, Taraboulos A, Stahl N, Prusiner SB (1990) Scrapie and cellular prion proteins differ in their kinetics of synthesis and topology in cultured cells. J Cell Biol 110: 743–752. 1968466 10.1083/jcb.110.3.743 PMC2116048

[9] Caughey B, Raymond GJ (1991) The scrapie-associated form of PrP is made from a cell surface precursor that is both protease- and phospholipase-sensitive. J Biol Chem 266: 18217–18223. 1680859

[10] Prusiner SB (2012) A unifying role for prions in neurodegenerative diseases. Science 336: 1511–1513. 22723400 10.1126/science.1222951 PMC3942086

[11] Collinge J, Clarke AR (2007) A general model of prion strains and their pathogenicity. Science 318: 930–936. 17991853 10.1126/science.1138718

[12] Telling GC, Parchi P, DeArmond SJ, Cortelli P, Montagna P, et al (1996) Evidence for the conformation of the pathologic isoform of the prion protein enciphering and propagating prion diversity. Science 274: 2079–2082. 8953038 10.1126/science.274.5295.2079

[13] Silveira JR, Raymond GJ, Hughson AG, Race RE, Sim VL, et al (2005) The most infectious prion protein particles. Nature 437: 257–261. 16148934 10.1038/nature03989 PMC1513539

[14] Smirnovas V, Baron GS, Offerdahl DK, Raymond GJ, Caughey B, et al (2011) Structural organization of brain-derived mammalian prions examined by hydrogen-deuterium exchange. Nat Struct Mol Biol 18: 504–506. 21441913 10.1038/nsmb.2035 PMC3379881

[15] Gambetti P, Cali I, Notari S, Kong Q, Zou W-Q, et al (2011) Molecular biology and pathology of prion strains in sporadic human prion diseases. Acta Neuropathol 121: 79–90. 21058033 10.1007/s00401-010-0761-3 PMC3077936

[16] Deleault NR, Walsh DJ, Piro JR, Wang F, Wang X, et al (2012) Cofactor molecules maintain infectious conformation and restrict strain properties in purified prions. Proc Natl Acad Sci U S A 109: E1938–46. 22711839 10.1073/pnas.1206999109 PMC3396481

[17] Kaneko K, Zulianello L, Scott M, Cooper CM, Wallace AC, et al (1997) Evidence for protein X binding to a discontinuous epitope on the cellular prion protein during scrapie prion propagation. Proc Natl Acad Sci U S A 94: 10069–10074. 9294164 10.1073/pnas.94.19.10069 PMC23307

[18] Perrier V, Kaneko K, Safar J, Vergara J, Tremblay P, et al (2002) Dominant-negative inhibition of prion replication in transgenic mice. Proc Natl Acad Sci U S A 99: 13079–13084. 12271119 10.1073/pnas.182425299 PMC130589

[19] Hölscher C, Delius H, Bürkle A (1998) Overexpression of Nonconvertible PrP c Δ 114–121 in Scrapie-Infected Mouse Neuroblastoma Cells Leads to trans -Dominant Inhibition of Wild-Type PrP Sc Accumulation Overexpression of Nonconvertible PrP c Δ 114–121 in Scrapie-Infected Mouse Neuroblastom. J Virol 72: 1153–1159. 9445012 10.1128/jvi.72.2.1153-1159.1998 PMC124590

[20] Shibuya S, Higuchi J, Shin RW, Tateishi J, Kitamoto T (1998) Codon 219 Lys allele of PRNP is not found in sporadic Creutzfeldt-Jakob disease. Ann Neurol 43: 826–828. 9629853 10.1002/ana.410430618

[21] Goldmann W, Hunter N, Foster JD, Salbaum JM, Beyreuther K, et al (1990) Two alleles of a neural protein gene linked to scrapie in sheep. Proc Natl Acad Sci U S A 87: 2476–2480. 1969635 10.1073/pnas.87.7.2476 PMC53712

[22] Palmer MS, Dryden AJ, Hughes JT, Collinge J (1991) Homozygous prion protein genotype predisposes to sporadic Creutzfeldt–Jakob disease. Nature 352: 340–342. 1677164 10.1038/352340a0

[23] Green KM, Browning SR, Seward TS, Jewell JE, Ross DL, et al (2008) The elk PRNP codon 132 polymorphism controls cervid and scrapie prion propagation. J Gen Virol 89: 598–608. 18198392 10.1099/vir.0.83168-0

[24] Mead S, Whitfield J, Poulter M, Shah P, Uphill J, et al (2009) A novel protective prion protein variant that colocalizes with kuru exposure. N Engl J Med 361: 2056–2065. 19923577 10.1056/NEJMoa0809716

[25] Geoghegan JC, Miller MB, Kwak AH, Harris BT, Supattapone S (2009) Trans-Dominant Inhibition of Prion Propagation In Vitro Is Not Mediated by an Accessory Cofactor. PLoS Pathog 5: e1000535. 19649330 10.1371/journal.ppat.1000535 PMC2713408

[26] Geissen M, Mella H, Saalmüller A, Eiden M, Proft J, et al (2009) Inhibition of Prion Amplification by Expression of Dominant Inhibitory Mutants - A Systematic Insertion Mutagenesis Study. Infect Disord Drug Targets 9: 40–47. 19200014 10.2174/1871526510909010040

[27] Vorberg I, Chan K, Priola SA (2001) Deletion of β -Strand and α -Helix Secondary Structure in Normal Prion Protein Inhibits Formation of Its Protease-Resistant Isoform. J Virol 75: 10024–10032. 11581371 10.1128/JVI.75.21.10024-10032.2001 PMC114577

[28] Lee CI, Yang Q, Perrier V, Baskakov I V (2007) The dominant-negative effect of the Q218K variant of the prion protein does not require protein X. Protein Sci 16: 2166–2173. 17766375 10.1110/ps.072954607 PMC2204135

[29] Westergard L, Turnbaugh JA, Harris DA (2011) A Nine Amino Acid Domain Is Essential for Mutant Prion Protein Toxicity. J Neurosci 31: 14005–14017. 21957261 10.1523/JNEUROSCI.1243-11.2011 PMC3227396

[30] Sigurdson CJ, Nilsson KPR, Hornemann S, Heikenwalder M, Manco G, et al (2009) De novo generation of a transmissible spongiform encephalopathy by mouse transgenesis. Proc Natl Acad Sci U S A 106: 304–309. 19073920 10.1073/pnas.0810680105 PMC2629180

[31] Sigurdson CJ, Nilsson KPR, Hornemann S, Manco G, Fernández-borges N, et al (2010) A molecular switch controls interspecies prion disease transmission in mice. J Clin Invest 120: 2590–2599. 20551516 10.1172/JCI42051 PMC2898603

[32] Hafner-Bratkovic I, Bester R, Pristovsek P, Gaedtke L, Veranic P, et al (2011) Globular domain of the prion protein needs to be unlocked by domain swapping to support prion protein conversion. J Biol Chem 286: 12149–12156. 21324909 10.1074/jbc.M110.213926 PMC3069419

[33] Ertmer A, Gilch S, Yun S-W, Flechsig E, Klebl B, et al (2004) The tyrosine kinase inhibitor STI571 induces cellular clearance of PrPSc in prion-infected cells. J Biol Chem 279: 41918–41927. 15247213 10.1074/jbc.M405652200

[34] Heiseke A, Aguib Y, Riemer C, Baier M, Schätzl HM (2009) Lithium induces clearance of protease resistant prion protein in prion-infected cells by induction of autophagy. J Neurochem 109: 25–34. 19183256 10.1111/j.1471-4159.2009.05906.x

[35] Gilch S, Bach C, Lutzny G, Vorberg I, Schätzl HM (2009) Inhibition of cholesterol recycling impairs cellular PrP(Sc) propagation. Cell Mol Life Sci 66: 3979–3991. 19823766 10.1007/s00018-009-0158-4 PMC2777232

[36] Bach C, Gilch S, Rost R, Greenwood AD, Horsch M, et al (2009) Prion-induced activation of cholesterogenic gene expression by Srebp2 in neuronal cells. J Biol Chem 284: 31260–31269. 19748890 10.1074/jbc.M109.004382 PMC2781524

[37] Supattapone S, Bosque P, Muramoto T, Wille H, Aagaard C, et al (1999) Prion Protein of 106 Residues Creates an Artificial Transmission Barrier for Prion Replication in Transgenic Mice. Cell 96: 869–878. 10102274 10.1016/s0092-8674(00)80596-6

[38] Winklhofer KF, Heske J, Heller U, Reintjes A, Muranyi W, et al (2003) Determinants of the in Vivo Folding of the Prion Protein. J Biol Chem 278: 14961–14970. 12556465 10.1074/jbc.M209942200

[39] Muramoto T, Scott M, Cohen FE, Prusiner SB (1996) Recombinant scrapie-like prion protein of 106 amino acids is soluble. Proc Natl Acad Sci U S A 93: 15457–15462. 8986833 10.1073/pnas.93.26.15457 PMC26426

[40] Supattapone S, Bouzamondo E, Ball HL, Wille H, Nguyen HB, et al (2001) A Protease-Resistant 61-Residue Prion Peptide Causes Neurodegeneration in Transgenic Mice. Mol Cell Biol 21: 2608–2616. 11259607 10.1128/MCB.21.7.2608-2616.2001 PMC86891

[41] Baskakov IV, Aagaard C, Mehlhorn I, Wille H, Groth D, et al (2000) Self-assembly of recombinant prion protein of 106 residues. Biochemistry 39: 2792–2804. 10704232 10.1021/bi9923353

[42] Chesebro B, Trifilo M, Race R, Meade-White K, Teng C, et al (2005) Anchorless prion protein results in infectious amyloid disease without clinical scrapie. Science 308: 1435–1439. 15933194 10.1126/science.1110837

[43] Baron GS, Wehrly K, Dorward DW, Chesebro B, Caughey B (2002) Conversion of raft associated prion protein to the protease-resistant state requires insertion of PrP-res (PrP(Sc)) into contiguous membranes. EMBO J 21: 1031–1040. 11867531 10.1093/emboj/21.5.1031 PMC125906

[44] Nishina KA, Supattapone S (2007) Immunodetection of glycophosphatidylinositol-anchored proteins following treatment with phospholipase C. Anal Biochem 363: 318–320. 17321480 10.1016/j.ab.2007.01.032 PMC1868555

[45] Barth S, Glick D, Macleod KF (2010) Autophagy: assays and artifacts. J Pathol 221: 117–124. 20225337 10.1002/path.2694 PMC2989884

[46] Ashok A, Hegde RS (2008) Retrotranslocation of Prion Proteins from the Endoplasmic Reticulum by Preventing GPI Signal Transamidation. Mol Biol Cell 19: 3463–3476. 18508914 10.1091/mbc.E08-01-0087 PMC2488287

[47] Ashok A, Hegde RS (2009) Selective Processing and Metabolism of Disease-Causing Mutant Prion Proteins. PLoS Pathog 5: e1000479. 19543376 10.1371/journal.ppat.1000479 PMC2691595

[48] Taraboulos A, Serban D, Prusiner SB (1990) Scrapie prion proteins accumulate in the cytoplasm of persistently infected cultured cells. J Cell Biol 110: 2117–2132. 1693623 10.1083/jcb.110.6.2117 PMC2116143

[49] Zulianello L, Kaneko K, Scott M, Erpel S, Han D, et al (2000) Dominant-Negative Inhibition of Prion Formation Diminished by Deletion Mutagenesis of the Prion Protein. J Virol 74: 4351–4360. 10756050 10.1128/jvi.74.9.4351-4360.2000 PMC111952

[50] Xu Z, Prigent S, Deslys J-P, Rezaei H (2011) Dual conformation of H2H3 domain of prion protein in mammalian cells. J Biol Chem 286: 40060–40068. 21911495 10.1074/jbc.M111.275255 PMC3220554

[51] Horiuchi M, Priola SA, Chabry J, Caughey B (2000) Interactions between heterologous forms of prion protein: binding, inhibition of conversion, and species barriers. Proc Natl Acad Sci U S A 97: 5836–5841. 10811921 10.1073/pnas.110523897 PMC18520

[52] Turnbaugh JA, Unterberger U, Saá P, Massignan T, Fluharty BR, et al (2012) The N-terminal, polybasic region of PrPC dictates the efficiency of prion propagation by binding to PrPSc. J Neurosci 32: 8817–8830. 22745483 10.1523/JNEUROSCI.1103-12.2012 PMC3433751

[53] Miller MB, Geoghegan JC, Supattapone S (2011) Dissociation of Infectivity from Seeding Ability in Prions with Alternate Docking Mechanism. PLoS Pathog 7: e1002128. 21779169 10.1371/journal.ppat.1002128 PMC3136465

[54] Yoshikawa D, Yamaguchi N, Ishibashi D, Yamanaka H, Okimura N, et al (2008) Dominant-negative effects of the N-terminal half of prion protein on neurotoxicity of prion protein-like protein/doppel in mice. J Biol Chem 283: 24202–24211. 18562311 10.1074/jbc.M804212200 PMC3259768

[55] Flechsig E, Shmerling D, Hegyi I, Raeber AJ, Fischer M, et al (2000) Prion protein devoid of the octapeptide repeat region restores susceptibility to scrapie in PrP knockout mice. Neuron 27: 399–408. 10985358 10.1016/s0896-6273(00)00046-5

[56] Leliveld SR, Dame RT, Wuite GJL, Stitz L, Korth C (2006) The expanded octarepeat domain selectively binds prions and disrupts homomeric prion protein interactions. J Biol Chem 281: 3268–3275. 16352600 10.1074/jbc.M510606200

[57] Smirnovas V, Kim J-I, Lu X, Atarashi R, Caughey B, et al (2009) Distinct structures of scrapie prion protein (PrPSc)-seeded versus spontaneous recombinant prion protein fibrils revealed by hydrogen/deuterium exchange. J Biol Chem 284: 24233–24241. 19596861 10.1074/jbc.M109.036558 PMC2782017

[58] Cobb NJ, Sönnichsen FD, McHaourab H, Surewicz WK (2007) Molecular architecture of human prion protein amyloid: a parallel, in-register beta-structure. Proc Natl Acad Sci U S A 104: 18946–18951. 18025469 10.1073/pnas.0706522104 PMC2141888

[59] Tycko R, Savtchenko R, Ostapchenko VG, Makarava N, Baskakov IV (2010) The α-helical C-terminal domain of full-length recombinant PrP converts to an in-register parallel β-sheet structure in PrP fibrils: evidence from solid state nuclear magnetic resonance. Biochemistry 49: 9488–9497. 20925423 10.1021/bi1013134 PMC3025268

[60] Surewicz WK, Apostol MI (2011) Prion Protein and Its Conformational Conversion: A Structural Perspective. Top Curr Chem 305: 135–168. 21630136 10.1007/128_2011_165

[61] Adrover M, Pauwels K, Prigent S, De Chiara C, Xu Z, et al (2010) Prion fibrillization is mediated by a native structural element that comprises helices H2 and H3. J Biol Chem 285: 21004–21012. 20375014 10.1074/jbc.M110.111815 PMC2898372

[62] Gilch S, Winklhofer KF, Groschup MH, Nunziante M, Lucassen R, et al (2001) Intracellular re-routing of prion protein prevents propagation of PrP Sc and delays onset of prion disease. EMBO J 20: 3957–3966. 11483499 10.1093/emboj/20.15.3957 PMC149175

[63] Ogier-denis E, Bauvy C, Cluzeaud F, Vandewalle A, Codogno P (2000) Glucose persistence on high-mannose oligosaccharides selectively inhibits the macroautophagic sequestration of N-linked glycoproteins. Biochem J 345: 459–466. 10642502 PMC1220778

[64] Fujita E, Kouroku Y, Isoai A, Kumagai H, Misutani A, et al (2007) Two endoplasmic reticulum-associated degradation (ERAD) systems for the novel variant of the mutant dysferlin: ubiquitin/proteasome ERAD(I) and autophagy/lysosome ERAD(II). Hum Mol Genet 16: 618–629. 17331981 10.1093/hmg/ddm002

[65] Ishida Y, Yamamoto A, Kitamura A, Lamande SR, Yoshimori T, et al (2009) Autophagic Elimination of Misfolded Procollagen Aggregates in the Endoplasmic Reticulum as a Means of Cell Protection. Mol Biol Cell 20: 2744–2754. 19357194 10.1091/mbc.E08-11-1092 PMC2688553

[66] Klionsky DJ, Abdalla FC, Abeliovich H, Abraham RT, Acevedo-Arozena A, et al (2012) Guidelines for the use and interpretation of assays for monitoring autophagy. Autophagy 8: 445–544. 22966490 10.4161/auto.19496 PMC3404883

[67] Goold R, Rabbanian S, Sutton L, Andre R, Arora P, et al (2011) Rapid cell-surface prion protein conversion revealed using a novel cell system. Nat Commun 2: 281. 21505437 10.1038/ncomms1282 PMC3104518

[68] Borchelt DR, Taraboulos A, Prusiner SB (1992) Evidence for synthesis of scrapie prion proteins in the endocytic pathway. J Biol Chem 267: 16188–16199. 1353761

[69] Taguchi Y, Shi Z-D, Ruddy B, Dorward DW, Greene L, et al (2009) Specific biarsenical labeling of cell surface proteins allows fluorescent- and biotin-tagging of amyloid precursor protein and prion proteins. Mol Biol Cell 20: 233–244. 18987338 10.1091/mbc.E08-06-0635 PMC2613110

[70] Marijanovic Z, Caputo A, Campana V, Zurzolo C (2009) Identification of an intracellular site of prion conversion. PLoS Pathog 5: e1000426. 19424437 10.1371/journal.ppat.1000426 PMC2673690

[71] Yamasaki T, Suzuki A, Shimizu T, Watarai M, Hasebe R, et al (2012) Characterization of intracellular localization of PrP(Sc) in prion-infected cells using a mAb that recognizes the region consisting of aa 119–127 of mouse PrP. J Gen Virol 93: 668–680. 22090211 10.1099/vir.0.037101-0

[72] Béranger F, Mangé A, Goud B, Lehmann S (2002) Stimulation of PrP(C) retrograde transport toward the endoplasmic reticulum increases accumulation of PrP(Sc) in prion-infected cells. J Biol Chem 277: 38972–38977. 12163492 10.1074/jbc.M205110200

[73] Deleault NR, Harris BT, Rees JR, Supattapone S (2007) Formation of native prions from minimal components in vitro. Proc Natl Acad Sci U S A 104: 9741–9746. 17535913 10.1073/pnas.0702662104 PMC1887554

[74] Deleault NR, Kascsak R, Geoghegan JC, Supattapone S (2010) Species-dependent differences in cofactor utilization for formation of the protease-resistant prion protein in vitro. Biochemistry 49: 3928–3934. 20377181 10.1021/bi100370b PMC3021175

[75] Deleault NR, Piro JR, Walsh DJ, Wang F, Ma J, et al (2012) Isolation of phosphatidylethanolamine as a solitary cofactor for prion formation in the absence of nucleic acids. Proc Natl Acad Sci U S A 109: 8546–8551. 22586108 10.1073/pnas.1204498109 PMC3365173

[76] Ma J (2012) The Role of Cofactors in Prion Propagation and Infectivity. PLoS Pathog 8: e1002589. 22511864 10.1371/journal.ppat.1002589 PMC3325206

[77] Heiseke A, Aguib Y, Schatzl HM (2010) Autophagy, prion infection and their mutual interactions. Curr Issues Mol Biol 12: 87–97. 19767652

