# Supplementary figures and images for: Critical Significance of the Region between Helix 1 and 2 for Efficient Dominant-Negative Inhibition by Conversion-Incompetent Prion Protein
Source: PLoS Pathog. 2013 Jun 27;9(6):e1003466. doi: 10.1371/journal.ppat.1003466 (PMC3694865; doi:10.1371/journal.ppat.1003466)

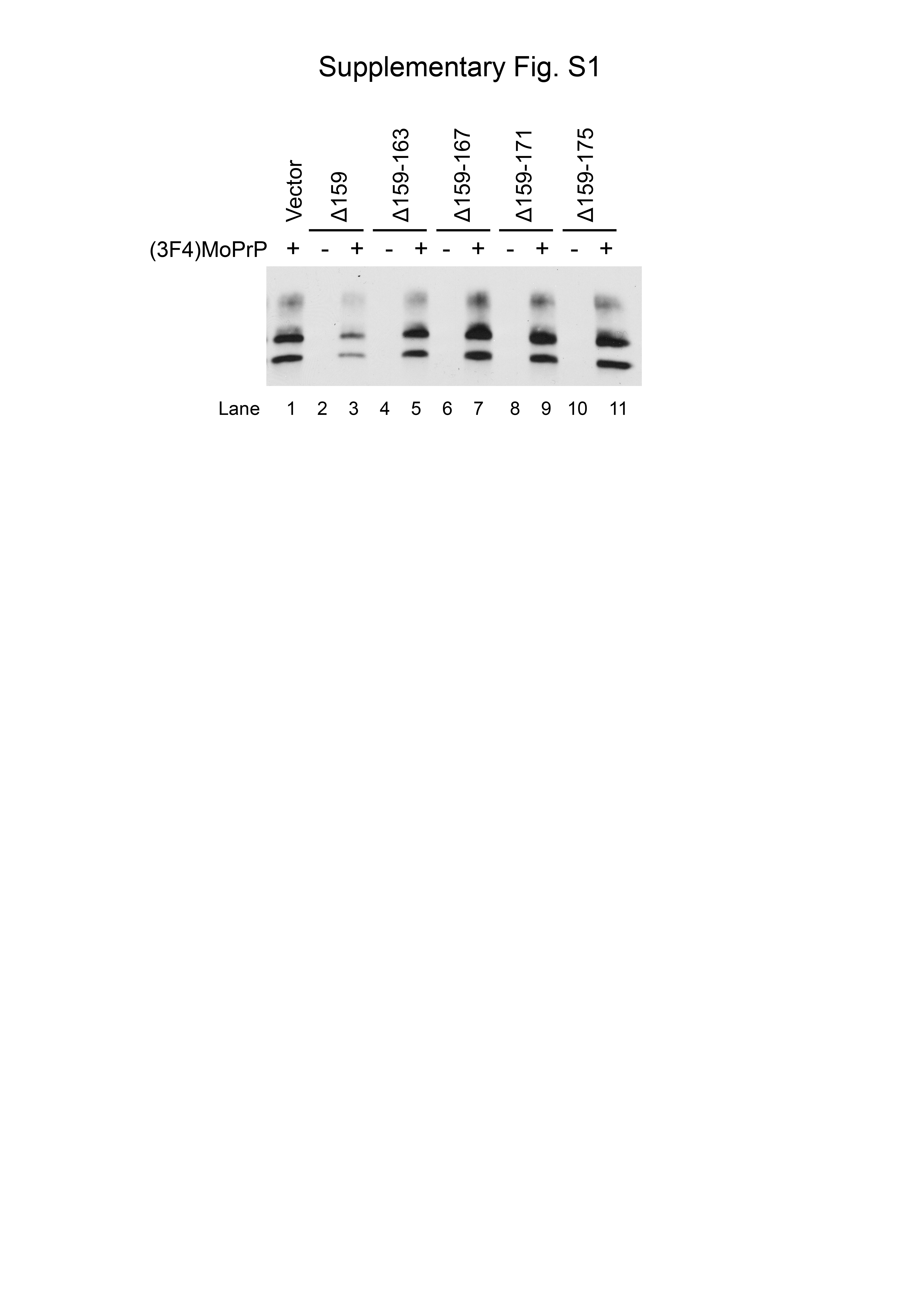

Supplement: Figure S1 — ΔPrPs are completely digested by PK at 25 µg/ml. Representative immunoblot probed with mAb 3F4 showing samples from 22L-ScN2a cells transiently transfected solely with plasmids encoding indicated ΔPrPs (even lanes) or co-transfected with mixture of same amounts of plasmids encoding respective ΔPrP and (3F4)MoPrP (odd lanes). Cells were harvested 24 hours after transfection and lysates digested with PK at 25 µg/ml for 30 minutes. Note that ΔPrPs were completely digested when transfected alone, proving that PK-resistant PrP detected in co-transfected cells represents solely those of (3F4)MoPrP. (TIF) [file ppat.1003466.s001.tif]

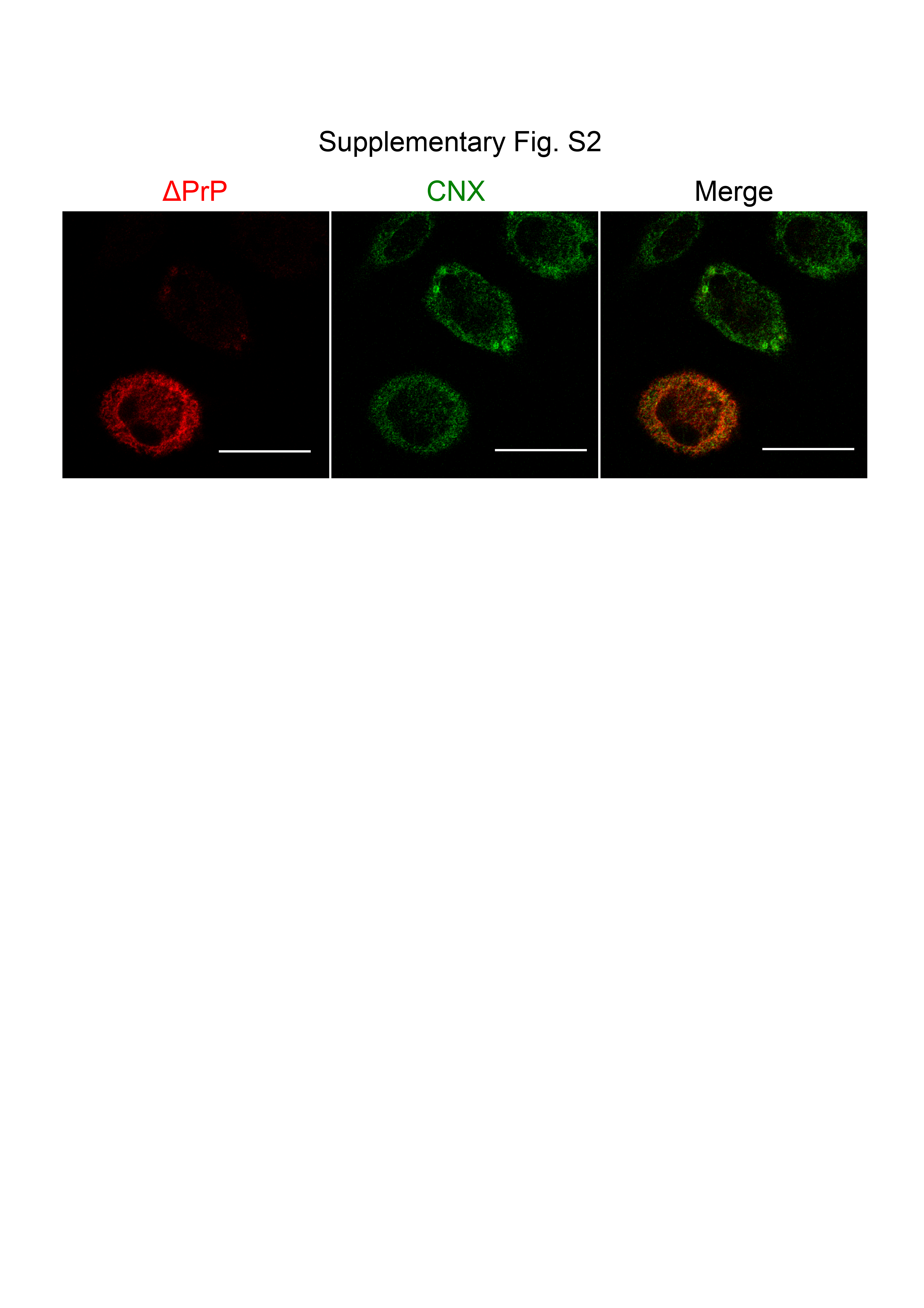

Supplement: Figure S2 — ΔPrP is moderately co-localized with calnexin (CNX). Confocal microscopy analysis of N2a cells transiently transfected with Δ159 and immuno-labeled with mAb 3F4 and anti-CNX polyclonal antibody. A section from the level of nuclei is shown. Note that both ΔPrP and CNX are diffusely distributed inside cells and show only a moderate degree of co-localization. Scale bar, 25 µm. (TIF) [file ppat.1003466.s002.tif]

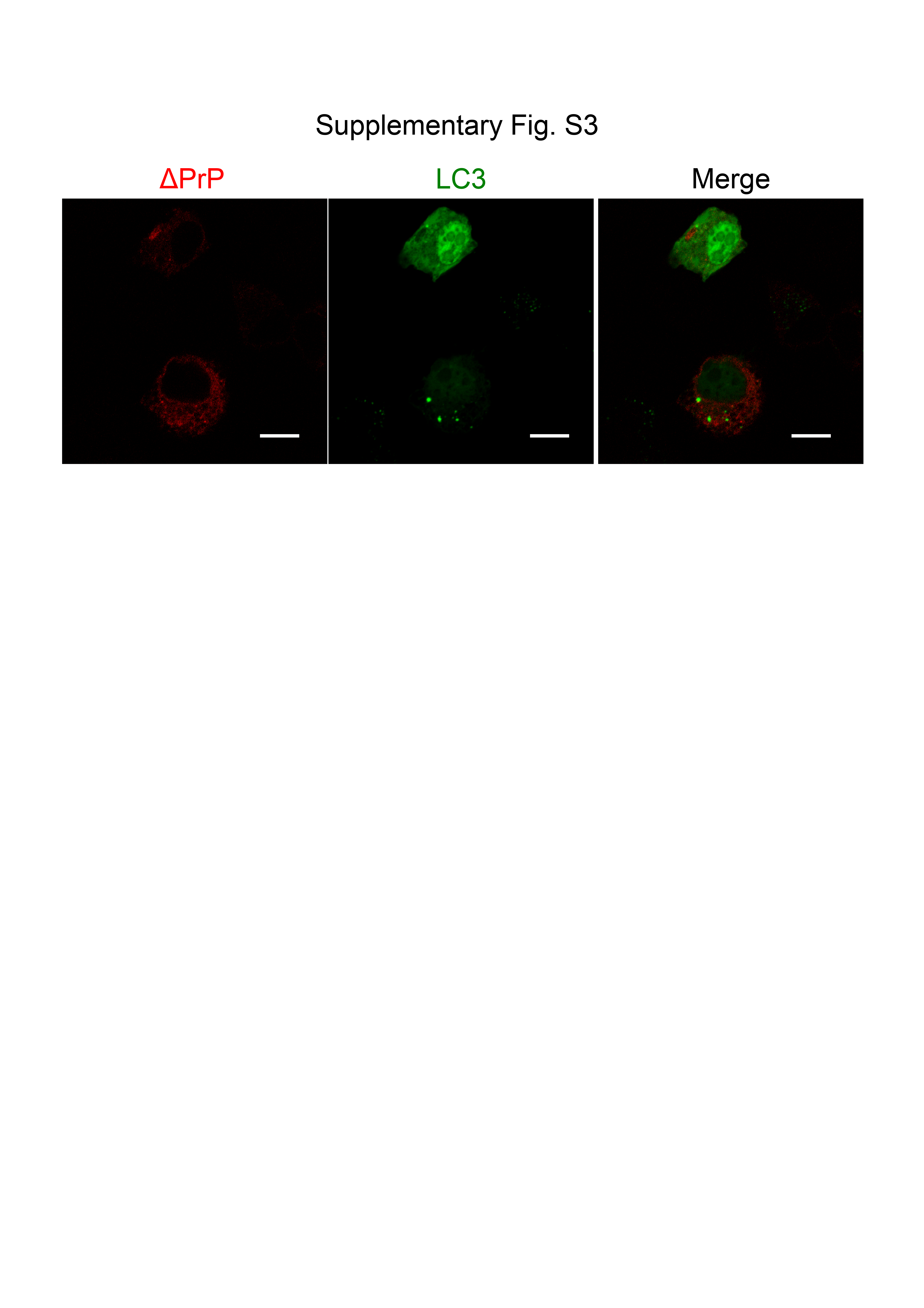

Supplement: Figure S3 — ΔPrP poorly co-localizes with EGFP-LC3 puncta. Confocal microscopy analysis of N2a cells transiently co-transfected with Δ159 and EGFP-LC3 and immuno-labeled with mAb 3F4. A section from the level of nuclei is shown, without GdnHCl treatment. Auto-fluorescent EGFP-LC3 puncta do not well co-localize with intensive fluorescent ΔPrP spots. Of note, this represents levels of basal autophagy without any induction of autophagy. Scale bar, 10 µm. (TIF) [file ppat.1003466.s003.tif]

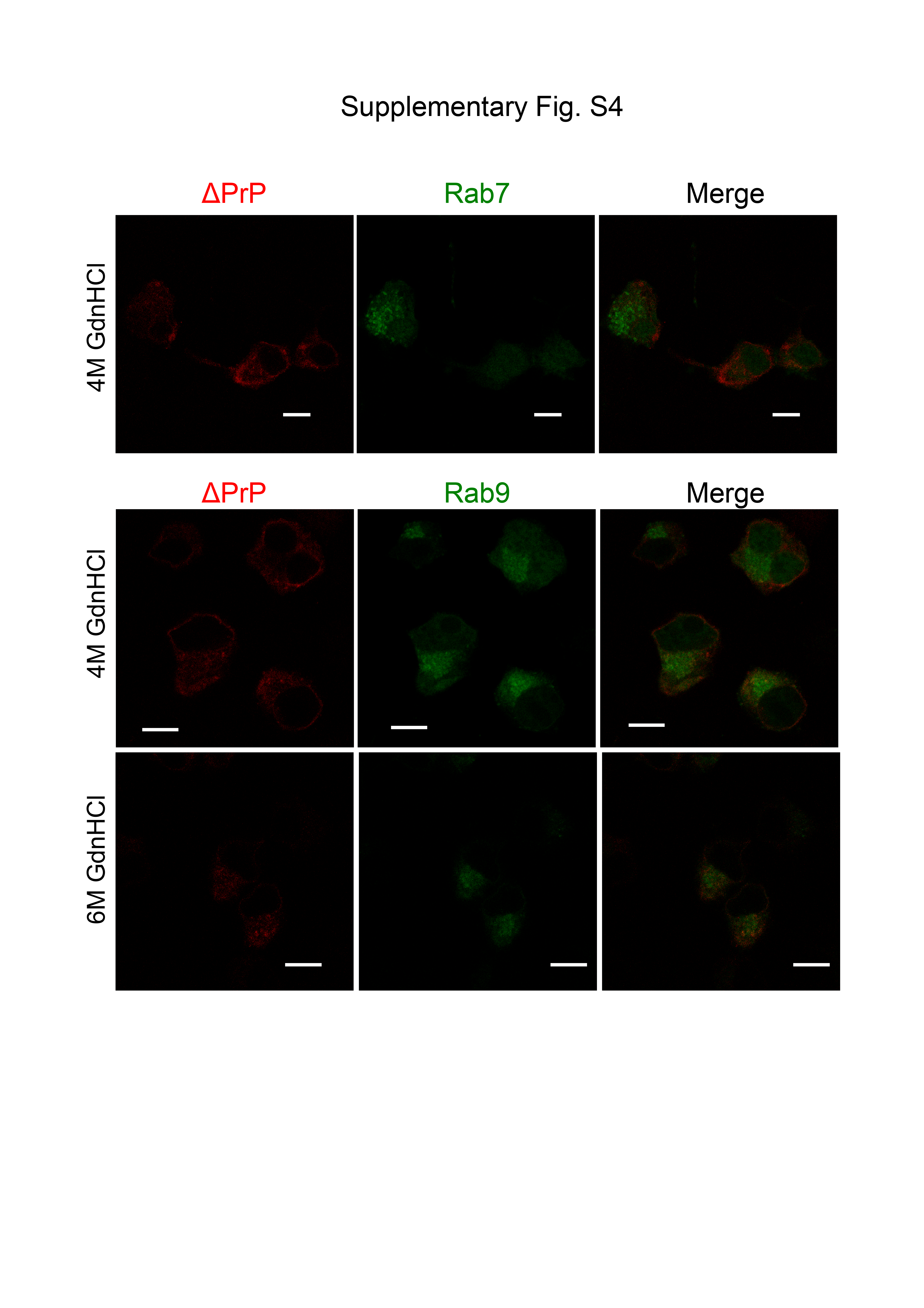

Supplement: Figure S4 — Attempts to observe co-localization of Δ159 with GFP-Rab7 or -Rab9. Confocal microscopy analysis of N2a cells co-transfected with Δ159 and EGFP-Rab7 or EGFP-Rab9. Transiently transfected N2a cells were fixed on cover slips and incubated with the indicated concentrations of GdnHCl for 45 minutes, followed by immuno-labeling with mAb 3F4 and DyLight594-conjugated anti-mouse IgG antibody. A section at the level of nuclei was used for co-localization analysis. EGFP-Rab7 and -Rab9 are found as blurry spots after GdnHCl treatment and a significant co-localization with 3F4-immunopositive structures is not found. Scale bars, 10 µm. (TIF) [file ppat.1003466.s004.tif]

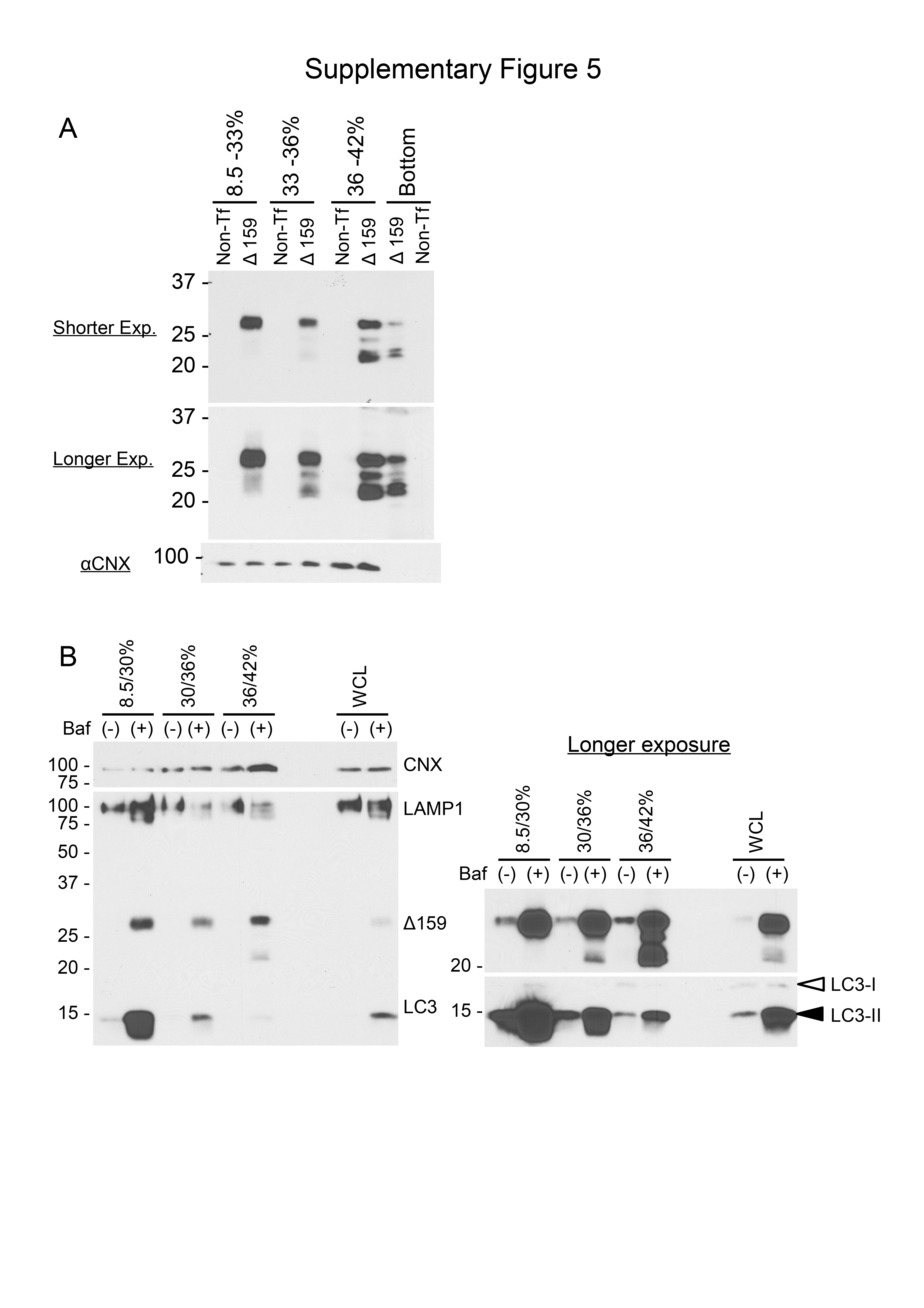

Supplement: Figure S5 — Subcellular fractionation provides further evidence for lysosomal degradation of Δ159. A. Subcellular fractionation on sucrose gradients consisting of 8.5%, 33% and 36% overlaid on homogenate (with 42% sucrose) from N2a cells with or without transfection of Δ159 (Non-Tf, non-transfected cells). A substantial amount of Δ159 is distributed to the 8.5%/33% interphase where calnexin (CNX) is relatively scarce (lower panel). Note that most of Δ159 in the 8.5%/33% interphase is diglycoform, while mono- and non-glycoforms are mainly present in the 36%/42% interphase where CNX is most enriched. B. Subcellular fractionation on sucrose gradients consisting of 8.5%, 30% and 36% overlaid on homogenate (with 42% sucrose) from transfected N2a cells with (Baf +) or without (Baf −) treatment with bafilomycin A1. Substantial amounts of Δ159 are reproducibly distributed to the low-density fraction (8.5%/30% interphase), where CNX is scarce and LAMP1 and LC3-II are most enriched. Amounts of the Δ159 diglycoform were strongly increased by bafilomycin A1 treatment. On the right a longer exposition is shown for PrP and LC3. The blot was simultaneously developed for PrP, LC3 and LAMP1, and then re-probed with anti-CNX antibody (αCNX). WCL, whole-cell lysates without fractionation. (TIF) [file ppat.1003466.s005.tif]
