# Supplementary material for: Critical Significance of the Region between Helix 1 and 2 for Efficient Dominant-Negative Inhibition by Conversion-Incompetent Prion Protein
Source: PLoS Pathog. 2013 Jun 27;9(6):e1003466. doi: 10.1371/journal.ppat.1003466 (PMC3694865; doi:10.1371/journal.ppat.1003466)
Supplement: Table S1 — Primers used for creating ΔPrPs. The term “Rev” in primer names indicates anti-sense primers. Antisense primer “Common Rev. Δ159-X” was combined with sense primers whose names start with “Δ159-” to create the internal deletions as indicated by the name of the sense primers by site-directed mutagenesis. Likewise, primer “Common ΔX-175” was combined with antisense primers whose names end with “-175” to create the internal deletions as indicated by their names. Δ31–160 was created by combining primers “ΔX-160” and “Rev. Δ31–160”. Δ171–175 was created by using primers “Δ171–175” and “RevΔ171–175”. Δ159–167(169) was accidentally created when engineering Δ159–167. (PDF) [file ppat.1003466.s006.pdf]

Table S1: Primers used to construct ΔPrPs.

Common Rev.Δ159-X	GTTAGGGTAGCGGTACATGTTTTCACGGTAGTAGCGGTCCTCCCA
Δ159	GTACCGCTACCCTAACGTGTACTACAGGCCAGTGGATCAGTACAGCAAC
Δ159-162	CATGTACCGCTACCCTAACAGGCCAGTGGATCAGTACAGC
Δ159-163	GTACCGCTACCCTAACCCAGTGGATCAGTACAGCAACCAGAACAACTTC
Δ159-164	CATGTACCGCTACCCTAACGTGGATCAGTACAGCAACCAG
Δ159-165	CATGTACCGCTACCCTAACGATCAGTACAGCAACCAGAAC
Δ159-167	GTACCGCTACCCTAACTACAGCAACCAGAACAACTTCGTGCACGACTGC
Δ159-171	GTACCGCTACCCTAACAACAACTTCGTGCACGACTGCGTCAATATCACC
Δ159-175	GTACCGCTACCCTAACCACGACTGCGTCAATATCACCATCAAGCAGCAC
Δ171-175	CAGTGGATCAGTACAGCAACCACGACTGCGTCAATATCACC
RevΔ171-175	GTTGCTGTACTGATCCACTGGCCTGTAGTACACTTGGTTAG
ΔX-160	TACTACAGGCCAGTGGATCAGTACAGCAACCAGAACAAC
Rev.Δ31-160	GATCCACTGGCCTGTAGTACCCTCCAGGCTTTGGCCGCTTG
Common ΔX-175	CACGACTGCGTCAATATCACCATCAAGCAGCACACGGTCACC
RevΔ171-175(2)	GATATTGACGCAGTCGTGGTTGCTGTACTGATCCACTGGCC
RevΔ31-175	GATATTGACGCAGTCGTGCCCTCCAGGCTTTGGCCGCTTC
RevΔ175	GATATTGACGCAGTCGTGGAAGTTGTTCTGGTTGCTGTACTG
